# Integrated miRNA/cytokine/chemokine profiling reveals severity-associated step changes and principal correlates of fatality in COVID-19

**DOI:** 10.1016/j.isci.2021.103672

**Published:** 2021-12-20

**Authors:** Julie C. Wilson, David Kealy, Sally R. James, Tobias Plowman, Katherine Newling, Christopher Jagger, Kara Filbey, Elizabeth R. Mann, Joanne E. Konkel, Madhvi Menon, Sean B. Knight, Angela Simpson, CIRCO Collaborative Group, Aliya Prihartadi, Greg Forshaw, Neil Todd, David R.A. Yates, John R. Grainger, Tracy Hussell, Paul M. Kaye, Nathalie Signoret, Dimitris Lagos

**Affiliations:** 1 Department of Mathematics, University of York, York YO10 5DD, UK; 2 Hull York Medical School, University of York, Wentworth Way, York YO10 5DD, UK; 3 York Biomedical Research Institute, University of York, York YO10 5DD, UK; 4 York Biosciences Technology Facility, University of York, Wentworth Way, York YO10 5DD, UK; 5 Lydia Becker Institute of Immunology and Inflammation, Division of Infection, Immunity & Respiratory Medicine, School of Biological Sciences, Faculty of Biology, Medicine and Health, University of Manchester, Manchester Academic Health Science Centre, Core Technology Facility, Room 2.16, 46 Grafton Street, Manchester M13 9PL, UK; 6 Maternal and Fetal Health Centre, Division of Developmental Biology, School of Medical Sciences, Faculty of Biology, Medicine and Health, University of Manchester, 5th Floor St. Mary's Hospital, Oxford Road, Manchester M13 9WL, UK; 7 Respiratory Department, Salford Royal NHS Foundation Trust, Stott Lane, Salford M6 8HD, UK; 8 Division of Infection, Immunity and Respiratory Medicine, Manchester NIHR BRC, Education and Research Centre, Wythenshawe Hospital, Manchester, UK; 9 The Members of the Coronavirus Immune Response and Clinical Outcomes (CIRCO) Collaborative Group; 10 York and Scarborough Teaching Hospitals NHS Foundation Trust, York YO31 8HE, UK

**Keywords:** Health sciences, Clinical finding, Complex system biology

## Abstract

Inflammatory cytokines and chemokines (CC) drive COVID-19 pathology. Yet, patients with similar circulating CC levels present with different disease severity. Here, we determined 171 microRNAomes from 58 hospitalized COVID-19 patients (Cohort 1) and levels of 25 cytokines and chemokines (CC) in the same samples. Combining microRNA (miRNA) and CC measurements allowed for discrimination of severe cases with greater accuracy than using miRNA or CC levels alone. Severity group-specific associations between miRNAs and COVID-19-associated CC (e.g., IL6, CCL20) or clinical hallmarks of COVID-19 (e.g., neutrophilia, hypoalbuminemia) separated patients with similar CC levels but different disease severity. Analysis of an independent cohort of 108 patients from a different center (Cohort 2) demonstrated feasibility of CC/miRNA profiling in leftover hospital blood samples with similar severe disease CC and miRNA profiles, and revealed CCL20, IL6, IL10, and miR-451a as key correlates of fatal COVID-19. These findings highlight that systemic miRNA/CC networks underpin severe COVID-19.

## Highlights

• MiRNA and CC profiles of 166 COVID-19 patients, including a leftover blood cohort
• Combining miRNA and CC profiling improves discrimination of severe COVID-19 cases
• Severity group-specific miRNA correlates of COVID-19-linked CC and clinical features
• 8 CC/3 miRNA signature of fatal COVID-19, including CCL20, IL6, IL10, and miR-451a

## Graphical abstract

**Figure:**
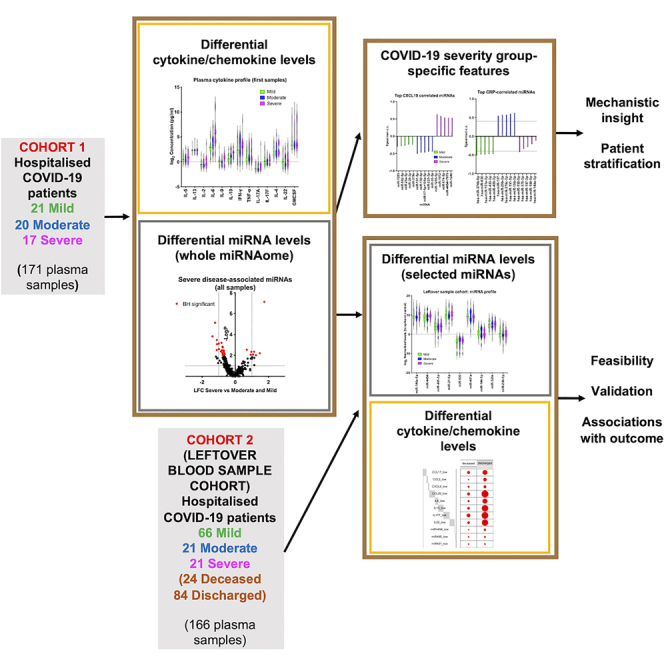

## Introduction

The COVID-19 pandemic has caused more than 5.2 million deaths (as of 1st December 2021, https://coronavirus.jhu.edu). More than 263 million people have been infected with SARS-CoV-2, the single-stranded RNA betacoronavirus that causes the disease. Infection results in a broad range of outcomes, from asymptomatic infection to lethal pneumonia, acute respiratory distress syndrome, and immunothrombosis (Chen et al., 2020a, 2020b). Significant disease heterogeneity is observed even within hospitalized individuals with COVID-19, with some requiring only management within a ward environment and modest supplemental oxygen support whereas others develop critical disease requiring invasive ventilation in intensive care units (ICUs). Age, comorbidities, host genetics, and the type of immune response are all thought to play a decisive role in shaping the trajectory of the infection (Esai Selvan, 2020).

There is a precarious balance between protective immunity and immune-driven pathology in COVID-19, with numerous studies identifying systemic and tissue correlates of severe and fatal disease. Systemic clinical and immune correlates of severity include elevated CRP and D-dimer levels, increased numbers of neutrophils, lymphopenia, T cell exhaustion, increased number of proliferating plasmablasts and extrafollicular B cell activation, and generation of platelet-sequestering nonclassical monocytes (Chen et al., 2020a; Diao et al., 2020; Schulte-Schrepping et al., 2020; Terpos et al., 2020; Vanderbeke et al., 2021; Woodruff et al., 2020; Wu et al., 2020). Consistent with the acute respiratory distress syndrome (ARDS) presentation of severe COVID-19, multiple studies have also reported increased circulating levels of pro-inflammatory cytokines and chemokines (CC) associated with COVID-19 severity including IL6, IL10, CCL2, CXCL10, and GMCSF (COvid-19 Multi-omics Blood ATlas (COMBAT) Consortium, 2021; Del Valle et al., 2020; Laing et al., 2020; Mann et al., 2020; Thwaites et al., 2021; Zhao et al., 2020). Other CC, have also been reported to predict severity, although less consistently (Chi et al., 2020; COvid-19 Multi-omics Blood ATlas (COMBAT) Consortium, 2021; Del Valle et al., 2020; Laing et al., 2020; Lucas et al., 2020; Zhao et al., 2020). However, it is notable that in the above studies a significant overlap in levels of COVID-19-associated CC is observed between disease severity groups.

Circulating microRNAs (miRNAs) have been shown to be excellent disease diagnostic or prognostic biomarkers in a wide range of chronic and acute inflammatory and infectious diseases including viral respiratory infection. Crucially, circulating miRNA levels are thought to reflect the state of the diseased tissue (Correia et al., 2017; Sohel, 2020; Wittmann and Jack, 2010; Zeng et al., 2014). Despite their proven value as mechanism-based clinical stratification indicators, miRNAs have only started being explored in the context of COVID-19. It has been proposed that miRNAs might be involved in controlling inflammation during COVID-19 (Aslani et al., 2021; Gasparello et al., 2021; Houshmandfar et al., 2021; Matarese et al., 2020; Plowman and Lagos, 2021). Theoretical CC/miRNA regulatory networks have been proposed but not validated (Khokhar et al., 2022), whereas the potential of miRNA-like therapeutics has also been suggested (Ahn et al., 2021; Dash et al., 2021; Hum et al., 2021; Park et al., 2021). Of note, initial studies have identified potential changes in miRNA expression in COVID-19 and signatures from targeted testing of 41 miRNAs have been linked to ICU treatment and mortality in a Spanish cohort (de Gonzalo-Calvo et al., 2021). Furthermore, it has been reported that suppression of the hsa-miR-320 family is linked to COVID-19 severity (Duecker et al., 2021). Correlations between specific miRNAs and response to treatment (Sabbatinelli et al., 2021) or D-dimer levels and risk of thrombosis in COVID-19 patients have also been proposed (Gambardella et al., 2021b). In addition, hsa-miR-2392 has been proposed both as a biomarker and therapeutic target of SARS-CoV-2 infection (McDonald et al., 2021).

We hypothesized that combining measurements of circulating CC and miRNAs would reveal qualitative changes associated with increased COVID-19 severity, leading to insight into determinants of COVID-19 outcome. We aimed to explore whether integrating miRNA with CC profiles would reveal a set of more accurate correlates of COVID-19 severity and outcome, and to identify severity-specific correlations of miRNAs with cytokine/chemokine and clinical parameter profiles in COVID-19. To achieve this, we measured CC levels as potential initiators of pathogenic signaling and circulating miRNAs as indicators of intracellular gene networks operating in combination with or in response to these CC. We first performed cytokine, chemokine, and miRNA profiling in 171 blood plasma samples from 58 hospitalized patients with mild, moderate, or severe COVID-19 (Cohort 1). We found that integrating clinical parameters with systemic correlates of immune response and tissue status (cytokines, chemokines, and miRNAs) revealed step changes occurring between clinical severity stages in COVID-19 and novel correlates of severe COVID-19. These severity group-specific qualitative features of COVID-19 were unique to each severity group and could only be observed by combining miRNA and CC profiling. Based on these findings, we analyzed 166 samples from a second cohort of 108 hospitalized individuals using blood samples that were leftover (or waste) from routine clinical testing following hospital admission (Cohort 2). Critically, we were able to detect CC and miRNAs in these samples with similar severe-disease associated factors. In this cohort, we were also able to search for principal systemic correlates of outcome (death or discharge). Again, this revealed that combinations of CC with miRNAs (e.g., CCL20, IL6, IL10, hsa-miR-451a) were better at predicting outcome than CC or miRNAs alone. Overall, our findings provide novel insight into trajectories underpinning severe disease in symptomatic COVID-19 patients and support that integrating miRNA and cytokine/chemokine profiling can shed light into determinants of disease severity as well as hematological and immunopathological features of COVID-19. Importantly, we demonstrate that miRNA/CC measurements can be reliably performed in leftover blood samples that can be easily accessed in any hospital setting.

## Results

### Severe COVID-19 is linked to distinct plasma miRNA signatures

We performed circulating miRNA profiling as a nucleic-acid-based approach to discovering peripheral correlates of disease in COVID-19 (Figure S1A). We analyzed samples from a first cohort (Cohort 1) of 58 hospitalized patients recruited between April and November 2020 in four hospitals in Manchester, UK as part of the CIRCO cohort (Table S1) (Mann et al., 2020). These samples were collected for research purposes. Patients were categorized in three groups of disease severity, mild (N = 20), moderate (N = 21), and severe (N = 17), based on the requirement for respiratory support as described before (Mann et al., 2020). Clinical measurements and comorbidity and symptom data were also available (Tables S2 and S3). For several patients, follow-up plasma samples were analyzed, collected at different time-points (typically taken every 1 to 2 days, Table S4).

**Figure 1:**
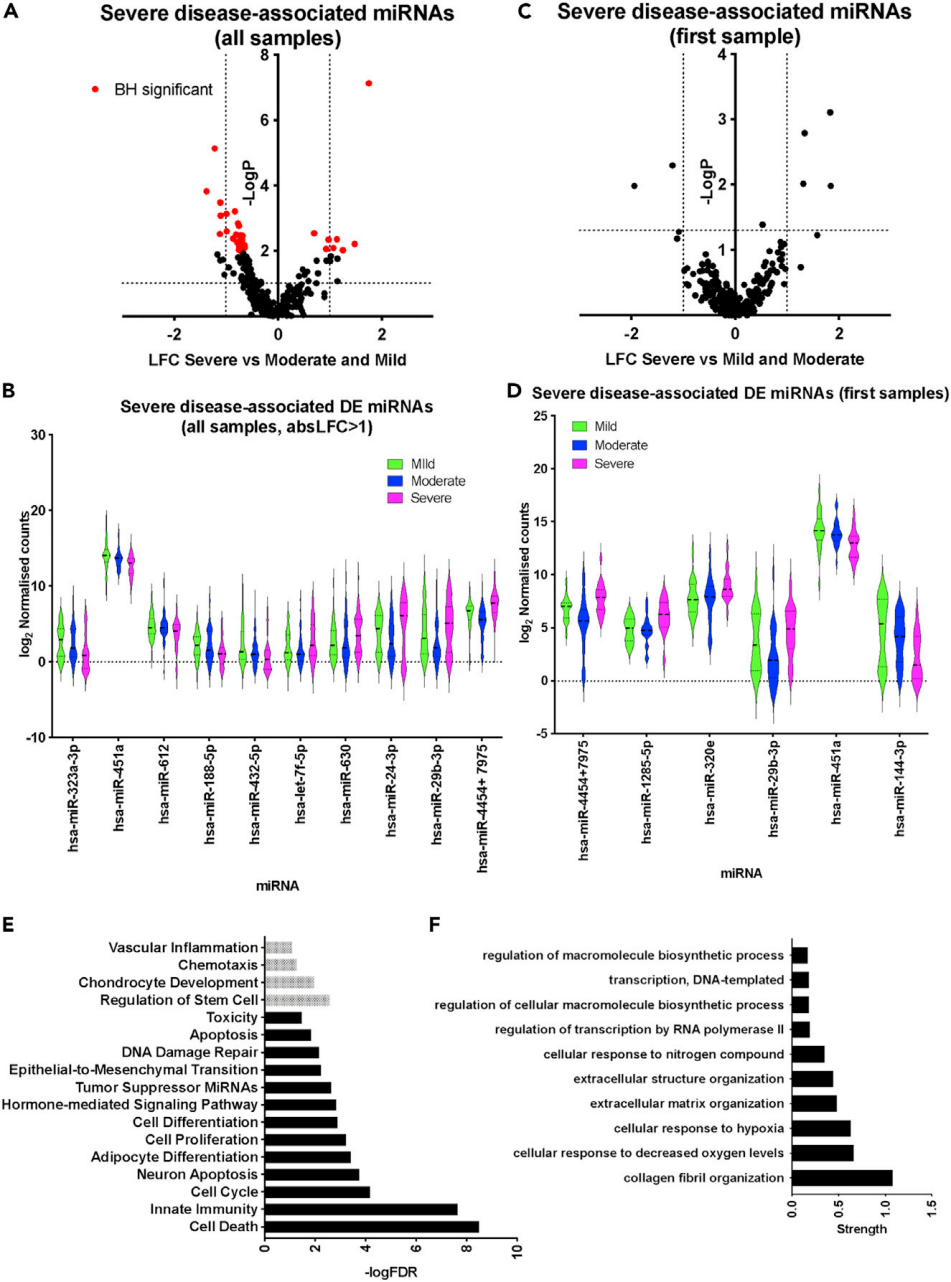
Plasma miRNA signatures of severe COVID-19 (A) Volcano plot comparing miRNA expression in severe against mild and moderate samples including all samples as independent observations. Dotted lines correspond to log_2_ fold change (LFC) greater than one and Mann Whitney p <0.05. Red dots correspond to miRNAs for which adjusted p <0.1 following Benjamini Hochberg (BH) correction. (B) Violin plots of top DE expressed miRNAs in severe samples (adjusted p <0.1 and absolute LFC>1), including all samples. (C) Volcano plot comparing miRNA expression in severe against mild and moderate samples including only the first available samples for each patient. Dotted lines correspond to log_2_ fold change (LFC) greater than one and Mann Whitney p <0.05. (D) Violin plots of DE expressed miRNAs in severe samples when only the first sample per patient is included (p <0.05 and absolute LFC>1). (E) Significantly overrepresented functional terms within upregulated (shown in black) and downregulated (shown in gray) miRNAs in severe cases (shown in red in (A)), including all samples as independent variables. (F) Significantly overrepresented gene ontology process terms among predicted targets of miRNAs upregulated in severe cases (red dots in the top right quadrant in (A).

Expression of miRNAs was determined by Nanostring profiling and 280 miRNAs were detected in at least 10% of the samples (see STAR Methods, Tables S5 and S6). First, we identified miRNAs that were differentially expressed in samples from individuals with severe disease vs the rest of the cohort (moderate and mild disease). When all samples were included in the analysis (N = 171 samples) we identified 32 significantly differentially expressed (DE) miRNAs (adjusted p <0.1; Figure 1A and Table S5). Downregulated miRNAs in severe disease samples (25 in total, Table S5) included hsa-miR-323a-3p, hsa-miR-451a, hsa-miR-188-5p, and hsa-miR-432-5p, whereas upregulated miRNAs (8 in total) included hsa-miR-4454 + 7975 (the mature sequence of hsa-miR-7975 differs only by one base from hsa-miR-4454), hsa-miR-let-7a-5p, hsa-miR-let-7f-5p, hsa-miR-630, hsa-miR-24-3p, and hsa-miR-29b-3p (Figure 1B and Table S5). We repeated the analysis including only the first available sample for each patient (N = 58 samples). Although, differences in miRNA expression did not reach significance after correcting for multiple testing, we identified six miRNAs which were DE in severe samples at p <0.05 and absolute log_2_ fold change (absLFC) > 1 (Figures 1C and 1D and Table S5). These were hsa-miR-4454 + 7975, hsa-miR-1285-5p, hsa-miR-320e, and hsa-miR-29b-3p, which were all upregulated, and hsa-miR-451a and hsa-miR-144-3p, which were downregulated. hsa-miR-4454 + 7975, hsa-miR-29b-3p, and hsa-miR-451a were identified in both analyses (including all samples or first samples). For these DE miRNAs, we did not observe any significant changes in expression over the duration of hospitalization (not shown). Having determined miRNA signatures associated with severe COVID-19 in hospitalized individuals, we performed functional association analysis using the TAM 2.0 tool (Li et al., 2018). Using the list of DE expressed miRNAs in severe cases (corresponding to Figure 1A), enriched functional terms for upregulated miRNAs included cell death, innate immunity, and cell cycle (Figure 1E). For downregulated DE miRNAs, enriched terms included Regulation of Stem Cell, Vascular inflammation, and Chemotaxis (Figure 1E). This indicated terms that were likely to reflect the immune response to infection (e.g., innate immunity) but also terms that could reflect tissue pathology (e.g., cell death). This was also the case, when performing functional term enrichment analysis using the list of all DE miRNAs based on first available samples only (corresponding to Figure 1B), with terms referring to wound healing and extracellular matrix remodeling being most enriched (Figure S1B). We note that statistical significance was lower because of the smaller number of DE miRNAs. We then explored functional associations of top predicted targets of upregulated miRNAs in severe samples using the Targetscan tool (Agarwal et al., 2015) (Figure S1C). Notably enriched gene ontology process associations included terms involving extracellular matrix remodeling and responses to hypoxia consistent with severe COVID-19 pathology (Figure 1F). Overall, our analyses revealed a miRNA signature associated with severe COVID-19, which was reflective of tissue pathology and damage.

### Progression from mild to moderate and severe COVID-19 is characterized by a global reduction in circulating miRNA levels

**Figure 2:**
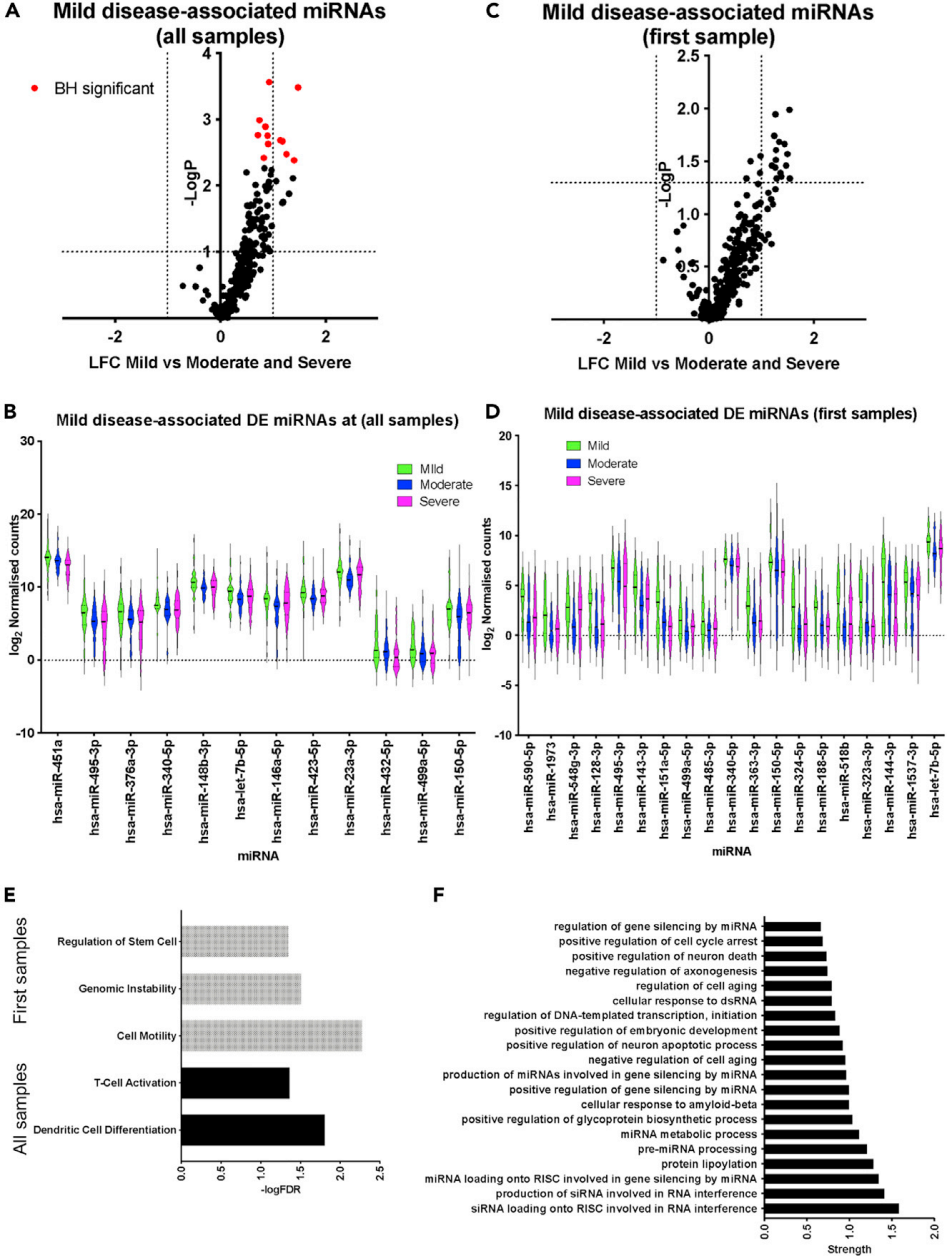
Plasma miRNA signatures of mild COVID-19 (A) Volcano plot comparing miRNA expression in mild against moderate and severe samples including all samples as independent observations. Dotted lines correspond to absolute LFC greater than one and Mann Whitney p <0.05. Red dots correspond to miRNAs for which adjusted p <0.1 following BH correction. (B) Violin plots of DE expressed miRNAs in mild samples (adjusted p <0.1), including all samples. (C) Volcano plot comparing miRNA expression in mild against moderate and severe samples including only the first available samples for each patient. Dotted lines correspond to absolute LFC greater than one and Mann Whitney p < 0.05. (D) Violin plots of DE expressed miRNAs in mild samples when only the first sample per patient is included (p <0.05 and absolute LFC >1). (E) Significantly overrepresented functional terms for miRNAs upregulated in mild samples when all samples (shown in black) or only first samples (shown in gray) are analyzed. (F) Significantly overrepresented gene ontology process terms among predicted targets of miRNAs upregulated in mild cases (red dots in the top right quadrant in (A).

Next, we explored miRNA signatures of mild COVID-19 disease within hospitalized individuals. When performing comparisons, either including all samples or just first available samples, the most notable finding was a decrease in circulating miRNA levels for the vast majority of detectable miRNAs in individuals with severe and moderate disease when compared to individuals with mild disease (Figures 2A–2D). When including all samples, mild cases were characterized by significantly higher levels (adjusted p <0.1) of miRNAs including hsa-miR-495-3p, hsa-miR-150-5p, hsa-miR-432-5p, hsa-miR-376a-3p, hsa-miR-146a-5p, and hsa-miR-451a (Figure 2B and Table S6). When including only the first available sample for each patient differences were more modest (p <0.05, absLFC>1), and DE miRNAs included hsa-miR-548g-3p, hsa-miR-150-5p, hsa-miR-590-5p, hsa-miR-518b, hsa-miR-363-3p, and hsa-miR-495-3p (Figures 2C and 2D, Table S6). Functional association analysis, using TAM 2.0, indicated an enrichment in miRNAs contributing to immune processes amongst DE miRNAs in mild COVID-19 (corresponding to Figure 2A), and more generic cellular processes when considering only the first samples (Figure 2E). Exploring gene ontology terms amongst the top predicted targets of upregulated miRNAs (Figure 2A), we observed enrichment in functional terms associated with mRNA and small RNA metabolism (Figure 2F). This could reflect the type response against SARS-CoV-2, a RNA virus. Alternatively, a change in RNA metabolism, including processing of small RNAs, could be associated with the higher circulating miRNA levels in patients with mild disease. Overall, these analyses revealed a miRNA signature associated with mild COVID-19, reflecting elements of both the immune response to SARS-CoV-2 infection but also tissue pathology.

### Cytokine and chemokine profiling reveal quantitative and qualitative differences between COVID-19 severity groups

**Figure 3:**
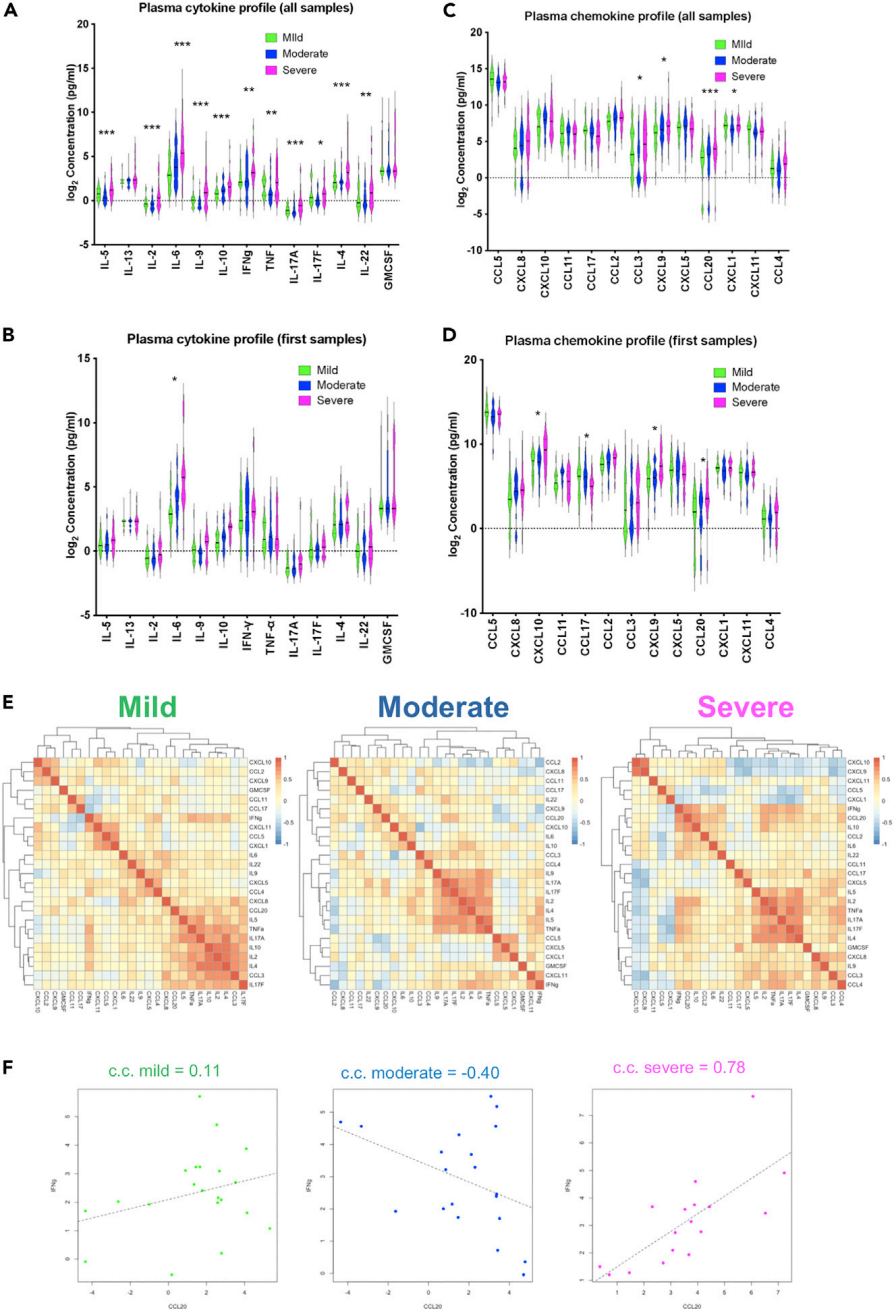
Circulating cytokine and chemokine signatures of COVID-19 (A) Violin plots of measured cytokines in mild (green), moderate (blue), and severe (magenta) samples, including all samples. Stars indicate significance for the comparison of severe against mild and moderate as follows: ∗ adjusted p <0.05, ∗∗ adjusted p <0.01, ∗∗∗ adjusted p <0.005. (B) As in A, but analysis includes only first available samples per patient. (C) As in A, but for circulating chemokines. (D) As in C, but analysis includes only first available samples per patient. (E) Heatmaps showing Spearman correlation between cytokines and chemokines for mild, moderate, and severe groups separately. The average value over all available measurements for each patient was used in the calculation of correlations. (F) Plots of IFNγ against CCL20 levels in mild (green), moderate (blue), and severe (magenta) patients. Correlation coefficients (c.c.) are shown above each scatter plot.

In addition to miRNA profiling, we measured cytokine (Figures 3A and 3B) and chemokine (Figures 3C and 3D) levels in the same plasma samples. When including all samples as independent variables, we observed significant (adjusted p <0.05) upregulation of most tested cytokines in severe samples (Figure 3A and Table S7). However, when taking only the first available samples for each patient only upregulation of IL-6 reached statistical significance when comparing severe samples to moderate and mild (Figure 3B). With regards to circulating chemokine levels, CCL3, CXCL9, CCL20, and CXCL1 were significantly upregulated in samples from individuals with severe COVID-19 when compared against mild and moderate cases (Figure 3C). Repeating the analysis to only include first samples, showed again increased levels of CXCL9 and CCL20 in severe disease, but also increased levels of CXCL10 and a downregulation of CCL17 in patients with severe COVID-19 (Figure 3D). Comparison of mild samples against the rest of the cohort (moderate and severe) revealed significantly lower levels of CXCL9, CCL20, IL6, IL10, IFNγ, and IL4 in mild samples, whereas levels of CCL5 were significantly higher (Table S7). Within the studied sampling time frame we did not observe any statistically significant patterns of regulation for DE CC over time (not shown).

We performed correlation analysis between individual CC, taking each sample as an independent observation (Figure S2A) or only including the first available sample for each patient (Figure S2B). However, we also performed analyses using the average levels for each patient. This approach has the benefit of capturing a representative and stable cytokine profile per patient, by including multiple sampling points for most patients (Table S4) but avoiding overrepresentation of some patients because of a higher number of collected samples. The abovementioned lack of significant changes in CC levels over the studied period meant that by taking an average of all measurements we were less likely to misrepresent the levels of any specific protein in the correlation matrix. Following this approach, we found that IL2, IL4, IL17A, IL17F, and TNF were positively correlated in all three severity groups (Figures 3E and S2C), as were IL5 and IFNγ but to a lesser extent particularly in the moderate group (Figures 3E, S2A, and S2B). Notably, IL10 was also part of this correlation cluster but only for patients with mild disease. Furthermore, CCL5 and CXCL1 were positively correlated in all groups. CXCL11 and CXCL5 were positively correlated with these, but only in mild and moderate disease groups respectively. CXCL10 and CXCL9 also correlated in all groups, although the correlation was stronger in severe cases (Figure 3E and S2D). IL9 was positively correlated with CCL3, CCL4, and CXCL8 in severe samples but to a lesser extent, or not at all in moderate and mild samples, respectively (Figure 3E). CXCL1 and IL17F were negatively correlated only in moderate and severe samples, the effect being stronger in severe. The same was observed for CXCL10 and CCL17. Notably, we found a strong qualitative difference between the three severity groups with regards to correlation between CCL20 and IFNγ, which were not correlated in mild, negatively correlated in moderate, and positively correlated in severe (Figure 3F). To identify relationships between severity groups and high or low levels of cytokines or chemokines, we first performed correspondence analysis with count data obtained by assigning values as low, medium, or high for each severity group (see STAR Methods). This showed that, elevated IL6 (p <0.001), IL9 (p <0.05), IL10 (p <0.01), and CXCL1 (p <0.05) were significantly associated with the severe disease group, whereas high values of CCL5 were associated with mild samples (Figure S2E). Overall, the CC profiling presented here and correlation analyses per severity group demonstrated that in addition to quantitative, there were also qualitative changes between severity groups.

### Integration of miRNA and CC profiles reveals severity group-specific circulating molecular signatures of COVID-19

**Figure 4:**
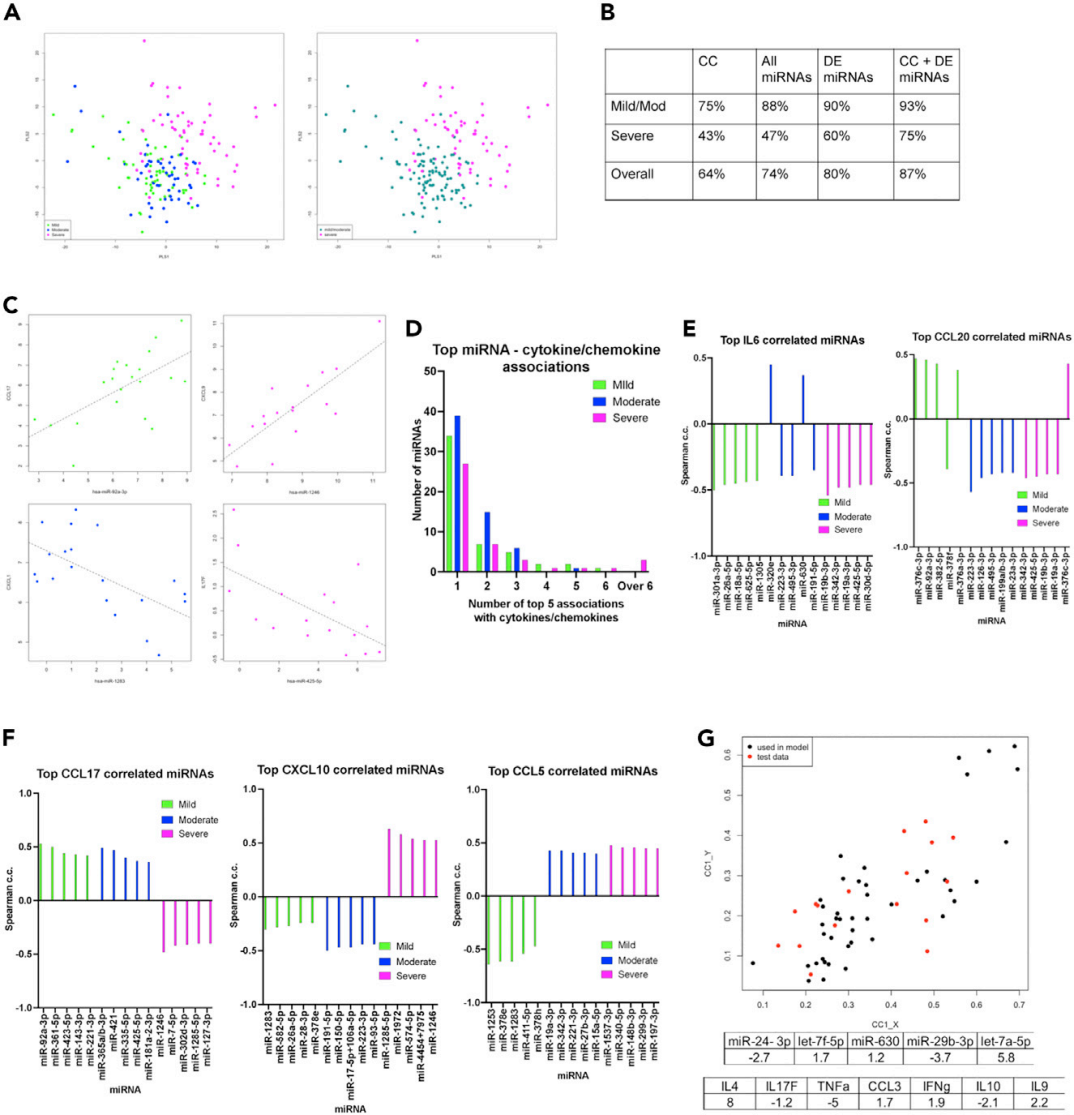
Integration of miRNA, and cytokine and chemokine signatures (A) Score plots for the first two latent variables from PLSR obtained using cytokine and chemokine data together with DE miRNA measurements. The plot on the left was obtained using all three severity levels (mild, moderate, and severe), whereas that on the right was obtained using only two classes (severe and mild/moderate). (B) Results from PLSR with patients considered in two groups for different datasets with continuous output values assigned to the nearest discrete class number. Accuracies shown were obtained using leave-one-patient-out cross validation, using only cytokine and chemokine (CC) values, all miRNA values, only DE miRNAs values, or combining all CC values with DE miRNAs. (C) Example scatterplots showing strong, severity group-specific correlations in mild (green), moderate (blue), or severe (magenta) patients. (D) Number of miRNAs (Y axis) in the top 5 strongest correlations with 1, 2, 3, 4, 5, 6, or over 6 cytokines/chemokines per severity group. See Table S4 (E) Spearman correlation coefficients for top 5 correlated miRNAs with IL6 and CCL20 in mild (green), moderate (blue), and severe (magenta) groups. (F) As in E, but for CCL17, CXCL10, and CCL5. (G) Canonical correlation analysis (CCA) to relate the miRNAs associated with cell death in the severe group (Figure 1E) with cytokines and chemokines. Cytokines/chemokines with individual absolute correlation greater than 0.4 (for the severe group) with any of these miRNAs were included in the analysis. The canonical scores plot shows data (from individual samples) for the12 patients used to determine the canonical covariates (c.c. = 0.8, p = 4.8 × 10^−6^) in black. The coefficients obtained are shown in the table and are used to obtain the canonical scores for 5 randomly chosen patients reserved as an independent test set (plotted in red).

To gain more insight into differences between severity groups we performed partial least squares regression (PLSR) analysis on the miRNA and CC profiles together (including all samples as independent observations). We found separation between samples from patients with severe disease and those with mild or moderate disease (Figure 4A and S3A). Using PLS models, we performed leave-one-patient-out (L-O-P-O) classification to assess the ability to predict severity based on our dataset. As discrimination between mild and moderate samples was poor, we repeated the analysis with just two classes, severe vs mild/moderate. The analysis was performed using only CC data, all miRNAs, only DE miRNAs, or CC data together with DE miRNAs. We found that combining the CC levels with DE miRNAs resulted in the highest overall accuracy (87%) in severity classification (Figure 4B).

To prevent individual patients with multiple samples dominating the analysis, we calculated correlations using average values (one per patient). Notably, correlations between miRNAs and CC calculated across the whole cohort were generally weak (Figure S3B). However, when performing the analysis within severity groups stronger and distinct correlations were discovered (Figure S3C and Table S8). The number of these correlations was notably higher in the severe disease group (Figure S3C, miRNAs and CC that show at least one significant correlation with correlation coefficient higher than 0.4). We observed severity group-specific features. CXCL1 showed positive correlations with a high number of miRNAs in severe disease compared to mild and moderate. CXCL1 also clustered with CXCL9 and CXCL10 in samples with severe disease but not in samples with moderate disease. IL6 showed several positive correlations with miRNAs and clustered with IL10, CCL20, GMCSF, IL22, and IFNγ in severe samples, and this was not the case for moderate and mild groups (with the exemption of CCL20). Interestingly, IL17F showed predominantly negative correlations with miRNAs specifically in the severe disease group (Figure S3C). Strong, severity-group specific correlations between CCL17 and miR-92a-3p (mild), CXCL1 and miR-1283 (moderate), IL-17F and miR-425-5p (severe), and CXCL9 and miR-1246 (severe) were also observed (Figure 4C). It is of note that, although these correlations were severity group-specific, none of these miRNAs were significantly differentially expressed in severe or mild samples (Tables S5 and S6).

### Distinct miRNA correlates of CC expression underpin severe COVID-19

For differentially expressed CC in severe or mild samples we determined the five miRNAs with the highest absolute correlation coefficients per severity group (Table S8). In addition to severity group-specific top miRNA correlates of plasma CC levels, this analysis revealed a striking qualitative difference for the severe group, namely the increased number of miRNAs featuring as top correlating with multiple CC (Figure 4D). In severe patients, miR-30d-5p was in the top 5 most correlated miRNAs for 9 CC. Furthermore, hsa-miR-425-5p and hsa-miR-19a-3p feature as top correlates of 8 CC, with hsa-miR-19b-3p featuring a further 6 times (Table S8). There were no miRNAs in the top 5 lists of more than 6 CC in mild or moderate cases. Conversely just 27 miRNAs appeared only once as one of the top 5 strongest correlations in severe cases, the number increasing to 39 and 34 for moderate and mild cases, respectively (Figure 4D). Of note, we observed distinct top correlating miRNA for each severity group for all tested CC, including for example IL6 and CCL20, two cytokines strongly associated with COVID-19 severity in our data (Figure 4E). In addition to different miRNAs, we found several cytokines with opposing correlation trends. For example, all top strongest miRNA correlations for CCL17 were negative in the severe group but positive in the mild and moderate, the reverse being the case for CXCL10. Similarly, the mild group was characterized by all top CCL5 correlations being negative in contrast to moderate and severe groups (Figure 4F). Conversely, miRNAs showed group-specific correlation patterns. For example, hsa-miR-24-3p, which was upregulated in patients with severe disease in cohort 1 (Table S3), showed a negative correlation with TNF and IL4 in the severe cases group only (Table S8).

Interestingly, the above cross-correlation analysis with miRNA expression (Figure S3C) separated in a severity-dependent manner the IL2, IL4, IL17A, IL17F, TNF cluster found in Figure 3E (when only CC expression was considered). This suggested that co-expression of these pro-inflammatory cytokines might be associated with distinct immunological and/or tissue pathology effects in each subgroup. We further explored this by focusing on miRNAs that contributed to enrichment of the cell death functional association amongst DE miRNAs in severe samples (Figure 1E). These miRNAs were miR-24-3p, Let-7f-5p, miR-630, miR-29b-3p, and Let-7a-5p. We reasoned that a set of chemokines or cytokines that would correlate with these miRNAs as a group could potentially be primary candidates to be effectors of lung tissue damage. We used canonical correlation analysis (CCA) to relate a linear combination of the miRNAs associated with cell death with a linear combination of the CC that showed the highest correlations individually with at least one of these miRNAs in severe samples (Table S9). The coefficients in the linear combinations with maximal correlation, obtained using samples from only 12 of the 17 patients in the severe group, are given in Figure 4G. For this data, the correlation for this first canonical pair was 0.8 (p = 4.79 × 10^−6^), identifying IL4 and TNF as the predominant contributors to the correlation with cell death miRNAs, with minor contributions from IL17F, CCL3, IFNγ, IL9, and IL10. We note here that collinearity because of correlations within these CC may make individual contributions difficult to estimate. Applying the coefficients to the data for samples from the remaining 5 severe patients showed that the model generalizes to unseen data with the correlation over all severe samples being 0.76. The canonical variates are plotted for both training and test data in Figure 4G. The above findings identified systemic signatures that were both quantitatively and qualitatively different between COVID-19 severity groups.

### Integration of miRNA and CC profiles with clinical parameters in hospitalized COVID-19 patients

We then explored how these could potentially underpin clinical features of COVID-19 by correlating levels of CC with clinical measurements. Clinical measurements were acquired only at the time of first blood sampling, therefore for this analysis we used CC levels from the first available plasma sample for each participant.

**Figure 5:**
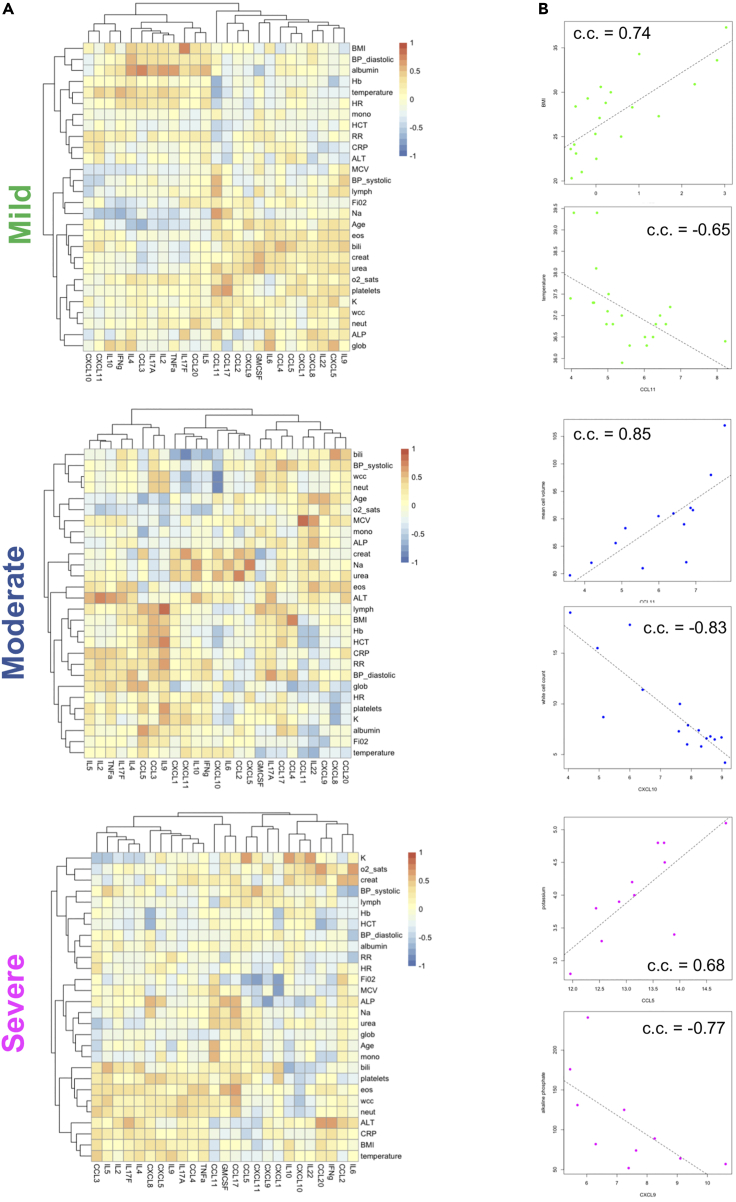
Correlation analysis of cytokine and chemokine profiles with clinical parameters (A) Heatmaps showing Spearman correlation coefficients (c.c.) between cytokines and chemokines and clinical measurements for mild, moderate, and severe groups. The first cytokine/chemokine value was used for each patient. (B) Example scatter plots for strong, severity group-specific correlations between cytokines/chemokines and clinical parameters in mild (green), moderate (blue), or severe (magenta) patients. Correlation coefficients (c.c.) are also shown.

In severe samples, we found a cluster including IL6, IFNγ, CCL2, and CCL20 positively correlating with ALT, CRP, saturated O_2_, and creatinine and negatively correlating with HCT (Figure 5A). A second cluster including TNF, IL17A, IL17F, IL9, IL2, and IL4 correlated positively with several cell populations including neutrophils and eosinophils but largely negatively with monocytes and lymphocytes. This clustering profile was specific to severe cases. Several strong and group-specific correlations were observed. IL9 correlated positively and strongly with lymphocytes in moderate cases, modestly in mild, and not at all in severe (Figure 5A). Other group-specific correlations included IL17F with BMI and CCL11 with temperature in mild, CCL11 with MCV and CXCL10 with WCC in moderate, and CCL5 with potassium and CXCL9 with ALP in severe (Figure 5B). Interestingly, we noted that cytokines widely associated with COVID-19 severity such IL6, CXCL10, and TNF showed multiple but modest correlations with clinical parameters in all groups, likely reflecting their multifunctional role in disease development.

**Figure 6:**
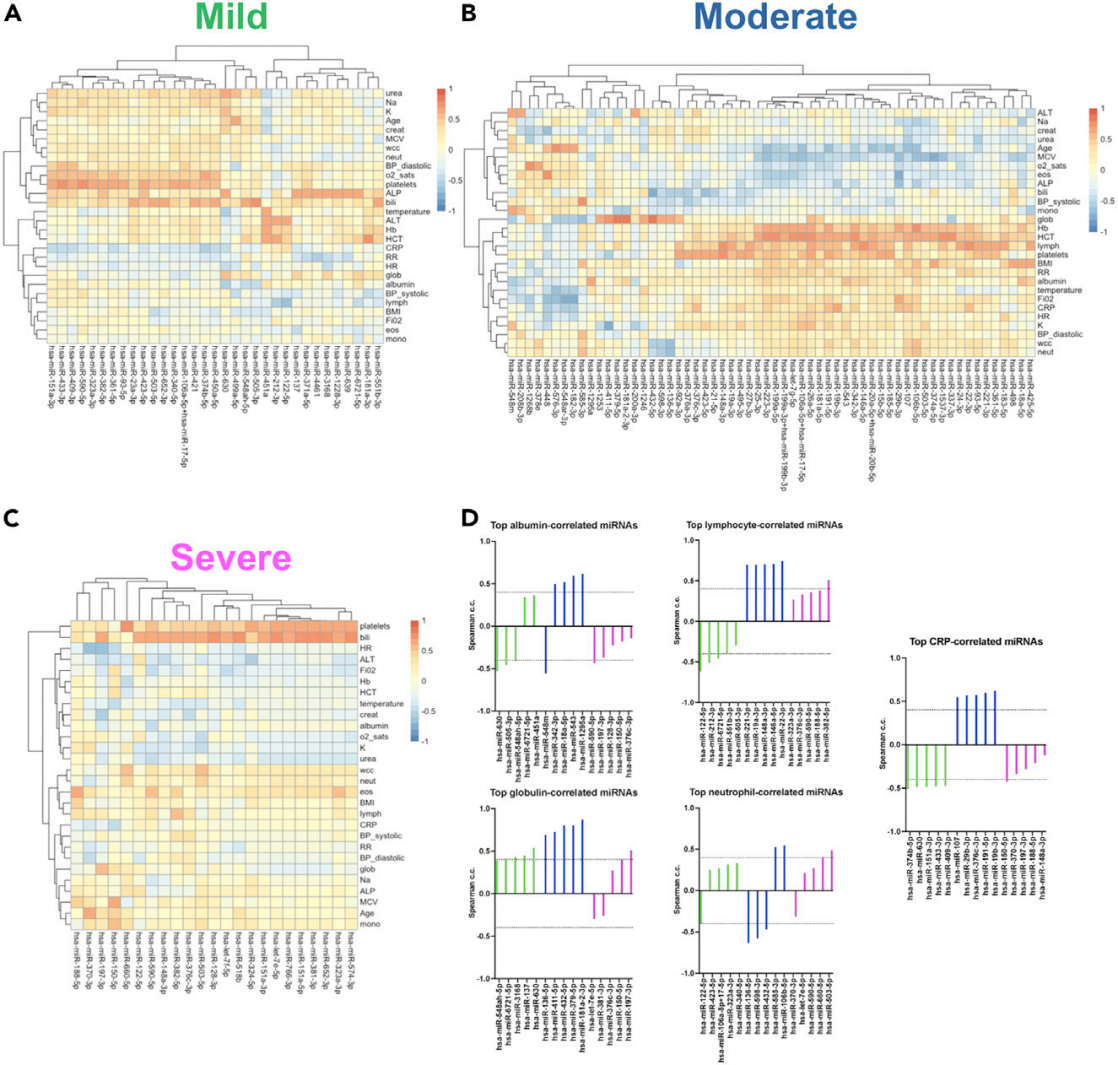
Severity-specific correlations between miRNAs and COVID-19-associated clinical parameters (A) Heatmap showing Spearman correlation coefficients (c.c.) between miRNAs and clinical measurements for the mild group of patients. Values shown for miRNAs with absolute c.c. >0.6 with at least one clinical parameter. The first miRNA value was used for each patient. (B) As in A, but for moderate cases. (C) As in A, but for severe cases. (D) Spearman correlation coefficients for top 5 correlated miRNAs with indicated clinical parameters in mild (green), moderate (blue), and severe (magenta) groups.

We also investigated significant correlations between levels of individual miRNAs and clinical measurements. The clinical parameters were separated in different clusters between groups and the number of strong correlations (absolute value >0.4) was higher in moderate cases (Figures 6A–6C). Of note clinical parameters, previously associated with COVID-19 severity, separated in different clusters between groups. For example, albumin and globulin (Feketea and Vlacha, 2020) clustered together in mild and moderate samples but not in severe (Figures 6A–6C) and the top miRNA correlates of albumin and globulin were distinct between severity groups (Figure 6D). In addition, the top miRNA correlates of lymphocyte numbers showed positive correlations in the moderate and severe groups but negative correlations in the mild, while we also observed severity group-specific correlations between miRNAs and neutrophils (Figure 6D). Interestingly, for CRP (Figure 6D) and hemoglobin (Figure S4), we found switches in the type of correlation and identity of miRNAs between mild and moderate cases, whereas only modest correlations were observed in the severe group (Figure 6C). Similar group-specific correlations were observed for all tested clinical parameters (Figure S4). In all groups we observed multiple positive correlations between platelets and individual miRNAs (Figure S4). However, we did not find any miRNAs that correlated with platelets in all groups. Eleven miRNAs strongly correlated with platelets in at least two groups. Overall, integration of cytokine, chemokine, and miRNA profiles with clinical parameters identified commonalities but also significant differences between disease severity groups in hospitalized individuals.

### Application of CC/miRNA profiling using leftover blood samples from routine clinical testing highlights factors associated with fatal COVID-19

**Figure 7:**
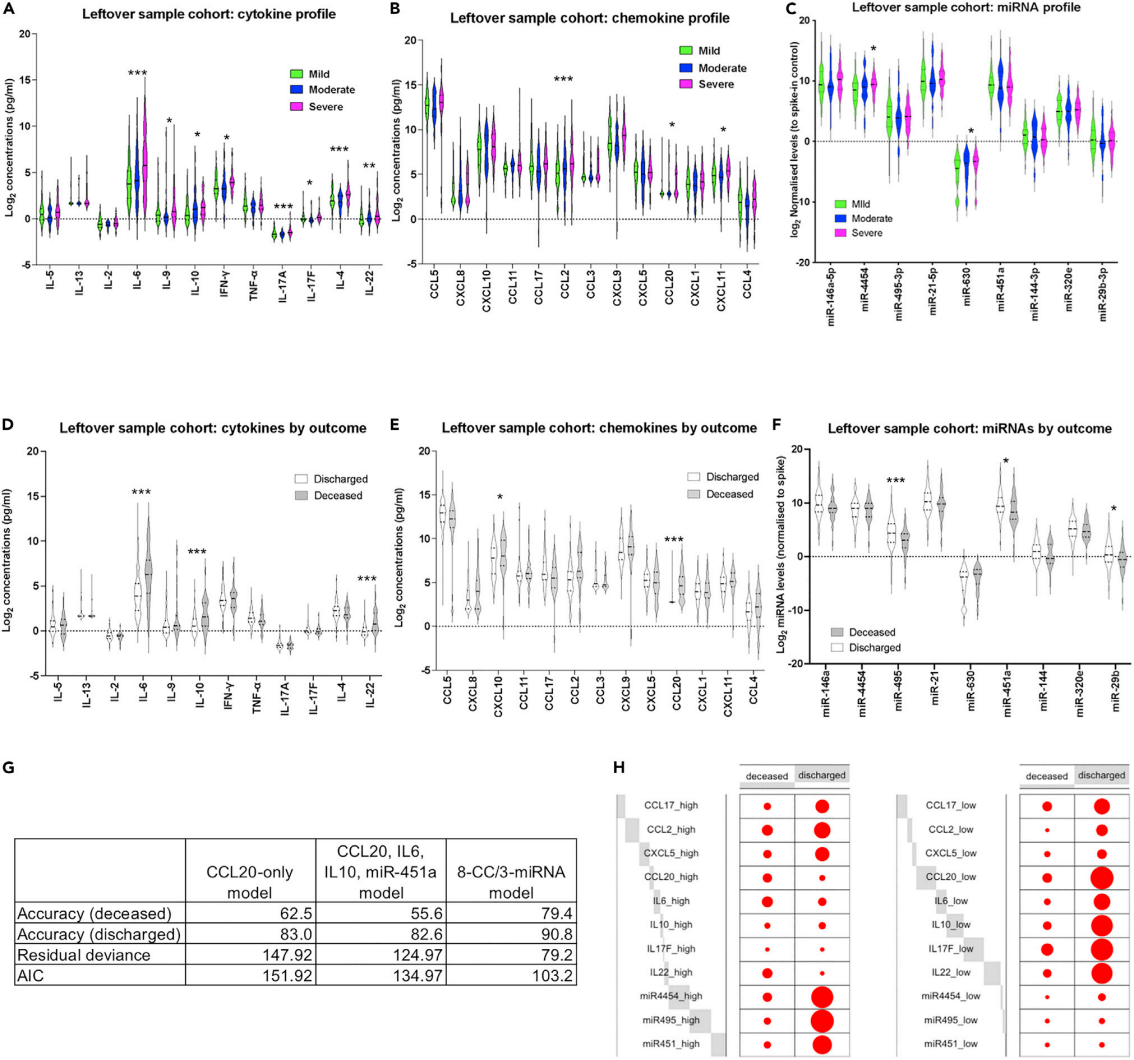
miRNA/CC correlates of fatal COVID-19 identified in a leftover blood sample cohort (A) Violin plots of measured cytokines in mild (green), moderate (blue), and severe (magenta) samples, in the leftover blood sample cohort. Stars indicate significance for the comparison of severe against mild and moderate as follows: ∗ adjusted p <0.05, ∗∗ adjusted p <0.01, ∗∗∗ adjusted p <0.005. (B) As in A, but for chemokine levels. (C) As in A, but for miRNA levels. (D) Violin plots of measured cytokines in samples from discharged (white) and deceased (gray) patients, in the leftover blood sample cohort. Stars indicate significance for the comparison of severe against mild and moderate as follows: ∗ adjusted p <0.05, ∗∗ adjusted p <0.01, ∗∗∗ adjusted p <0.005. (E) As in D, but for chemokine levels. (F) As in D, but for miRNA levels. (G) Results of forward regression models including accuracy for deceased and discharged patients, residual deviance, and AIC. Results are shown for the CCL20-only model, the CCL20/IL6/IL10/miR-451a model, and the 3 miRNAs/11 CC models (see Table S11). (H) Balloon plots for the parameters included in the 3 miRNAs/11 CC model. The size of each dot reflects the number of patients in the deceased or discharged group with high (left panel), or low (right panel) levels of the shown CCs and miRNAs. Gray blocks indicate the proportion of the total counts (high or low) each variable accounts for. See STAR methods for definition of high and low values.

To test the potential for wider application of miRNA/CC profiling in clinical practice, we analyzed an independent COVID-19 patient cohort including 166 leftover blood samples from 108 hospitalized individuals (66 mild, 21 moderate, 21 severe) between January and March 2020 (cohort 2). These samples were collected for clinical assessment and diagnostic purposes (e.g., complete blood counts). Importantly, samples had to be stored at room temperature (typically 12–48 h) before plasma separation, to be available in case they were required for further clinical tests. Samples were collected at admission for all patients, with some patients having follow-up samples (Table S10). In this cohort, we measured levels of miRNAs identified as differentially expressed in cohort 1 by qRTPCR, including hsa-miR-4454 (we chose this over hsa-miR-7975 as it is more abundant based on publicly available data in www.miRBase.org), hsa-miR-29b-3p, hsa-miR-451a, hsa-miR-630, hsa-miR-146a-5p, hsa-miR-495-3p, hsa-miR-320e, and hsa-miR-144-3p. We also measured levels of hsa-miR-21-5p as a control miRNA that did not show differential expression between groups in cohort 1 (Figures 1 and 2, and Tables S5 and S6). We opted to measure miRNA levels by qRTPCR as an orthogonal technique to Nanostring profiling, appropriate for candidate miRNA measurement. Levels of all CC shown in Figure 2 were also measured. Strikingly, despite storage of these samples at room temperature before plasma isolation, we observed notable similarities in differential expression. As in cohort 1, levels of CCL2, CCL20, IL6, IL9, IL10, IFNγ, IL17A, IL17F, IL4, IL22, hsa-miR-4454, and miR-630 were significantly (adjusted p <0.05) different in severe cases (Figures 7A–7C). It is of note that several cytokine and chemokine receptors are predicted targets of the two miRNAs found to be associated with severe disease in both cohorts. Predicted hsa-miR-4454 targets include IL10, IL1R1, IL13RA1, IL18BP, IL10RB, and CCR4, and predicted hsa-miR-630 targets include IL7, IL17D, IL23R, IL15RA, CCR4, CXCL13, CXCL14, CCR2, CCL11, and CCR5. We observed similar core correlations between CC (i.e., correlations occurring in all groups) as in cohort 1, including CXCL10 with CXCL9 (and CXCL11 in cohort 2), IL4, with IL71A, IL17F, and TNF (and IL2 to a lesser extent), CXCL1 with CXCL5, and IL6 with IL10 and IL22 (Figures S5A–S5C). Similarly to cohort 1, CXCL10 and CCL17 showed a negative correlation only in severe and moderate cases. We note that we did not observe a strong correlation between CCL20 and IFNγ in severe cases, although we still observed the strongest negative correlation between CCL20 and IFNγ in moderate cases as was the case with cohort 1 (compare Figure S5B and 3F). We did not perform correlation analyses between miRNAs and CC, as we only measured levels of 9 preselected miRNAs rather than the whole miRNome.

The higher number of patients in the cohort 2 allowed us to test CC and miRNA correlates of COVID-19 outcome (discharge or death). Twenty-four patients were deceased and 84 discharged (Table S10). We found that fatal COVID-19 was associated with higher levels of IL6, IL10, IL22, CXCL10, CCL2, and CCL20 and lower levels of miR-495-3p, miR-451a, and miR-29b-3p (Figures 7D–7F). To identify main systemic correlates of fatality, we used forwards stepwise logistic regression to construct models connecting miRNAs and CC with outcome. We started with a null model and allowed any of the variables found to be statistically significant individually (Figures 7A–7F) to be added if the model improved. This resulted in a model including CCL20, IL6, IL10, and hsa-miR-451a (Figures 7G and Table S11), which was compared to a model with only CCL20 (the most statistically significantly associated factor with fatal COVID-19, adjusted p = 1.02 × 10^9^). Although classification accuracies were comparable between the two models, both the residual deviance (the lack of fit to the perfect model) and the Akaike information criterion (AIC; a measure of prediction error) were smaller for this model, indicating that the CCL20/IL6/IL10/hsa-miR-451a model fitted the data better and suggesting a potential role for these CC and miRNA in fatal COVID-19. A third model that considered all variables without strong outliers (CXCL10, CCL17, CCL2, CCL3, CXCL9, CXCL5, CCL20, CXCL11, CCL4, IL6, IL10, TNF, IL17F, IL22, hsa-miR-4454, hsa-miR-29b-3p, hsa-miR-451a, hsa-miR-630, hsa-miR-146a-5p, hsa-miR-495-3p, and hsa-miR-144-3p) resulted in inclusion of 11 variables, including three miRNAs: CCL17, CCL2, CXCL5, CCL20, IL6, IL10, IL17F, IL22, hsa-miR-4454, hsa-miR-495-3p, hsa-miR-451a (Figures 7G and 7H, Table S11). This 3miRNA/11CC model showed the highest accuracy (79%–90%) and lowest deviance (79.204) and AIC (103.2). Taken together, the second cohort analyses demonstrated the feasibility of miRNA and CC profiling in leftover blood samples, identified CC and miRNAs that were statistically elevated in severe cases in both cohorts, and allowed us to build a model highlighting CC and miRNA correlates of fatal COVID-19.

## Discussion

Our study represents the largest circulating miRNA profiling study in COVID-19, including whole miRNAome and multiplex CC analysis of 337 samples from 166 patients from 4 hospitals and two different geographical locations in the UK. Using miRNA profiling to deconvolute CC abundance resulted in identifying sets of miRNAs that correlate in a severity-specific manner with CC already associated with COVID-19 severity such as IL6 and CXCL10 (Coperchini et al., 2021). Overall, we show that determining correlations between miRNAs and these CC can robustly identify patients with severe disease in a manner not possible through analysis of CC alone. Importantly, these miRNAs reflect intracellular gene networks (e.g., hypoxia, apoptosis, etc) providing mechanistic insight into the context-dependent function of CC in severe COVID-19. It is of note that several of the severity group-specific miRNA-CC or miRNA-clinical parameter correlations we report involve miRNAs that are not significantly differentially expressed in severe or mild samples. This indicates the emergence of disease-associated novel networks rather than just quantitative increase of interactions operating in a continuum during progression from mild to moderate to severe disease. Interestingly, although such qualitative changes are more numerous and profound when combining miRNA and CC profiling, a small number can be already determined when exploring CC levels alone. In Cohort 1, IFNγ and CCL20 showed a strong positive correlation only in patients with severe disease. Of note, IFNγ is an essential trigger of antimicrobial pulmonary responses, but increased IFNγ levels in COVID-19 blood and airways samples are associated with pathogenesis and pulmonary tissue damage (Chua et al., 2020; Karki et al., 2021; Montalvo Villalba et al., 2020), possibly through a detrimental effect of IFNγ on epithelial cells (Heuberger et al., 2021). On the other hand, CCL20 is secreted by the bronchial epithelium and important for lung immunity, but it can lead to pathogenic responses (Ito et al., 2011). Therefore, the strongly correlated IFNγ and CCL20 may mark a circulating readout of severe respiratory disease complications.

Our approach can be used as a foundation for stratifying patients and understanding response to CC-targeting treatments such as anti-IL6R blocking antibodies (Galvan-Roman et al., 2021). Indeed, small-scale studies have proposed candidate miRNA predictors of response to IL6R blockade (Sabbatinelli et al., 2021). In this respect, testing the applicability of our findings from Cohort 1 when analyzing leftover (or waste) blood samples (Cohort 2), which are obtained in every hospital as part of routine care, has wide-reaching implications in clinical practice. Identifying CC and miRNAs with altered levels in patients with severe COVID-19 in both cohorts is notable, as in addition to significant differences in method and time of sampling (e.g., immediate processing and freezing, Ficoll-based separation, collection within 1 to 2 days of admission for Cohort 1 vs storage at room temperature for 12 to 24 h, plasma isolation by centrifugation, and collection at hospital admission in Cohort 2), Cohort 1 was predominantly recruited during the first wave of the pandemic but Cohort 2 was recruited during the second wave of the pandemic in the UK. Therefore, commonalities between the two cohorts span different dominant SARS-CoV-2 variants and changes in clinical practice that occurred between the first two waves of the pandemic. Differences between the two cohorts should be interpreted cautiously as they can be a result of technical or immune-virological differences between the two cohorts.

A correlation between circulating and diseased tissue profiles has been extensively reported in cancer, and autoimmune and infectious diseases (Correia et al., 2017; Sohel, 2020; Wittmann and Jack, 2010; Zeng et al., 2014). Our analyses in Cohort 1 show that both circulating miRNAs and their predicted targets are indeed reflective of processes occurring at the tissue level (e.g., cell death, response to hypoxia, extracellular matrix remodeling). This provides a powerful tool in further understanding main activities of multifunctional CC. For example, we identify a significant linkage between IL4 and TNF and cell death-associated miRNAs, including hsa-miR-24-3p, which has previously been linked to cerebrovascular effects (Gambardella et al., 2021a; Mone et al., 2021). These associations do not imply causality, as changes in circulating CC levels can also be the consequence of tissue damage. Interestingly, hsa-miR-24-3p was also proposed as a potential component of computationally predicted miRNA/CC regulatory networks operating during COVID-19 (Khokhar et al., 2022), and was with hsa-miR-335-5p one of the only two miRNAs from this study to be highlighted in our analyses. In addition, a smaller study compared 10 COVID-19 patients with 10 healthy controls and identified 55 DE miRNAs in COVID-19 patients (Farr et al., 2021). Seven of these miRNAs (hsa-let-7a-5p, hsa-let-7f-5p, hsa-miR-150-5p, hsa-miR-423-5p, hsa-miR-432-5p, hsa-miR-451a, hsa-miR-651-5p) were also found to be associated with severity in our study, as they were DE in severe or mild patients. Another study reported down-regulation of hsa-miR-320a,b,c family members when comparing 10 severe cases to 8 healthy controls (Duecker et al., 2021), something we did not observe in our study. This discrepancy could be because of the small size of the previous study, the fact that the previous study compared COVID-19 patients to healthy controls using a different miRNA detection platform (Nanostring vs small RNA sequencing), or differential expression of family members between cohorts.

A strength of our analysis in Cohort 1 was the ability to perform correlation analysis with clinical laboratory measurements. In severe samples, two CC clusters were observed one of which (TNF, IL17A, IL17F, IL9, IL2, and IL4) correlated positively with neutrophils and largely negatively with monocytes and lymphocytes, providing insight into inflammatory profiles associated with lymphopenia and neutrophilia occurring in severe COVID-19 (Henry et al., 2020; Terpos et al., 2020). Of note, several miRNAs correlate with platelets in our analysis. Although we cannot exclude the possibility that this could be partly because of platelet contamination or lysis, among a list of 11 miRNAs previously reported to strongly correlate with platelet contamination (Mitchell et al., 2016) only two (hsa-miR-93-5p and hsa-miR-17-5p) correlated with platelets in more than one severity group in our study. Alternatively, as platelet-secreted miRNAs reflect platelet activation and function (Czajka et al., 2021), severity group-specific platelet-associated miRNA signatures could provide novel insight into the observed thrombocytopenia and platelet aggregation phenotype in severe COVID-19 (Lippi et al., 2020; Rampotas and Pavord, 2020).

Although Cohort 1 allowed us to perform unbiased miRNA and CC profiling in high-quality clinical samples, its smaller size meant that it was not possible to attempt analyses of outcome. Cohort 2, however, allowed us to perform this analysis. The two cohorts could not be merged because of the different methodologies used to measure miRNA levels. Others have reported a link between elevated IL6 and IL10 levels to fatality (Li et al., 2021) providing external validation of our data collected from leftover blood samples. However, these factors alone are not sufficient for prediction of outcome (Liu et al., 2021b). Our study also revealed CCL20 as a key correlate of fatal COVID-19. Increased circulating CCL20 levels in blood as well as respiratory tract samples have been reported in severe COVID-19 patients and linked to ARDS (Chua et al., 2020; Hue et al., 2020; Saris et al., 2021), but the profound link to fatal COVID-19 observed here is less appreciated. Importantly, combining miRNA with CC profiling leads to improved and more accurate models of correlates of fatal COVID-19 including hsa-miR-451a, hsa-miR-495-3p, and hsa-miR-4454. Of note hsa-miR-451a was also linked to COVID-19 in the above-mentioned comparison of COVID-19 patients to healthy controls (Farr et al., 2021). These findings provide a solid foundation for other studies further validating fatal COVID-19-associated CC and miRNAs.

Overall, we propose that integrated miRNA/CC profiling can determine key systemic changes that reflect the step-wise progression of COVID-19. Other methodologies such as whole blood RNA sequencing, single cell RNA sequencing, high-resolution proteomics, or autoantibody profiling also support the existence of distinct disease progression trajectories characterized by unique quantitative and qualitative features (Bergamaschi et al., 2021; Bost et al., 2020; Liu et al., 2021a; Stephenson et al., 2021; Wang et al., 2021). Identifying qualitative differences between different COVID-19 severity manifestations and quantitative correlates of potentially fatal disease in symptomatic individuals and investigating their mechanistic relevance can provide important clues for development of future COVID-19 therapies. Using exclusively circulating measurements and easy-to-obtain samples to dissect these differences means that our work already provides a set of candidate biomarkers that can be used by the clinical and research community to study pathogenesis and treatment of COVID-19. We propose that targeted tandem miRNA-CC profiling provides a flexible noninvasive toolkit to help mechanistic studies, patient stratification, and response-to-treatment monitoring for future and current COVID-19 therapeutics.

### Limitations of the study

One limitation of our study is that its size does not allow us to perform extensive analysis of all possible confounders (Tables S1 and S3) and risk analysis to identify independent predictors of COVID-19 severity or outcome. As such future studies in independent cohorts will need to further validate our results. Another limitation of our study is that only patients from the North of England were recruited. Although the severity-associated CC profiles identified here do not significantly differ to those reported in previous studies (COvid-19 Multi-omics Blood ATlas (COMBAT) Consortium, 2021; Del Valle et al., 2020; Laing et al., 2020; Mann et al., 2020; Thwaites et al., 2021; Zhao et al., 2020), this geographic limitation should be highlighted. In terms of other biases associated with observational cohort studies (Sedgwick, 2014), we note that although the number of follow-up samples was not different between severity groups (Table S4), the duration varied. To counter this bias, we performed some analyses including only the first available sample or the mean value from all samples for each participant. Furthermore, although we cannot exclude selection biases, such effects are likely to be reduced through including only hospitalized COVID-19 patients (all exposed and positive to SARS-CoV-2; no healthy controls) and studying two cohorts, one of which recruited prospectively. Lastly, as all our measurements were taken from plasma the cellular source of detected CC and miRNAs is not known, therefore it is not possible to assess whether there are direct interactions between the detected miRNAs and CC.

## STAR★Methods

### Key resources table

**Table:** 

REAGENT or RESOURCE	SOURCE	IDENTIFIER
**Biological samples**		

Plasma samples from COVID-19 hospitalised patients (cohort 1)	Manchester hospitals under the framework of the Manchester Allergy, Respiratory and Thoracic Surgery (ManARTS) and the Northern Care Alliance Research Collection (NCARC)	ManARTS Biobank (study no M2020-88, ethics: 15/NW/0409); NCARC tissue biobank (study no. NCA-009; ethics 18/WA/0368).
Leftover hospital blood samples from hospitalised COVID-19 patients (cohort 2)	York District Hospital, York and Scarborough NHS Trust	REC reference 19/YH/0394 with approved SA002 amendment of IRAS project 269597

**Critical commercial assays**		

LEGENDplex™ HU Th Cytokine Panel (12-plex) w/ VbP V02	BioLegend	Cat #741028
LEGENDplex Human Proinflammatory chemokine Panel 1	BioLegend	Cat #741081
LEGENDplex Human CCL5 1-plex	BioLegend	Cat #741083
GM-CSF ABTS ELISA kit	Peprotech	
Norgen Plasma/Serum RNA Purification Mini kit	Norgen	Cat #55000
miRNA Taqman assays	Thermo Fischer	Cat #4427975
Taqman MicroRNA Reverse Transcription Kit	Thermo Fischer	Cat #4366596
Taqman Universal PCR Master Mix	Thermo Fischer	Cat #4305719
Nanostring human v3 miRNA for 827 human miRNAs	Nanostring	CSO-MIR3-12

**Deposited data**		

Nanostring miRNA data	GEO	GSE189610

**Software and algorithms**		

R statistical programming language	The R project	https://r-project.org
Prism	GraphPad	https://graphpad.com

**Other**		

StepOnePlus Real Time PCR System	Thermofisher	Cat #4376600
nCounter® FLEX Analysis System – nCounter Prepstation	Nanostring Inc	NA
nCounter® FLEX Analysis System – nCounter Digital Analyser	Nanostring Inc	NA

### Resource availability

#### Lead contact

Requests for resources, datasets, and protocols, should be directed to and will be fulfilled by the lead contact, Dimitris Lagos (Dimitris.lagos@york.ac.uk).

#### Materials availability

This study did not generate new unique reagents.

### Experimental model and subject details

#### Clinical study design and sample collection

The clinical recruitment and study design for cohort 1 has been reported elsewhere (Mann et al., 2020). Briefly, individuals with COVID-19 were recruited from 3 hospitals in the Greater Manchester area, Manchester University Foundation Trust (MFT), Salford Royal NHS Foundation Trust (SRFT) and Pennine Acute NHS Trust (PAT) under the framework of the Manchester Allergy, Respiratory and Thoracic Surgery (ManARTS) Biobank (study no M2020-88) for MFT or the Northern Care Alliance Research Collection (NCARC) tissue biobank (study no. NCA-009) for SRFT and PAT. Ethics approval was obtained from the North West-Haydock Research Ethics Committee for ManARTS (reference 15/NW/0409) and from the Wales Research Ethics Committee 4 for NCARC (reference 18/WA/0368). Participants were admitted to hospital between April and November 2020 (Tables S1–S4). Samples were collected as soon after admission as possible and at one- or two-day intervals thereafter (Table S4).

For cohort 2, the leftover blood cohort, individuals with COVID-19 were recruited at York District Hospital, York and Scarborough NHS Trust between January and March 2020 (Table S10). Patients were recruited at admission. Ethics approval was obtained from Yorkshire & The Humber - Leeds West Research Ethics Committee (REC reference 19/YH/0394 with approved SA002 amendment of IRAS project 269597). Samples were first used for complete blood counts and stored for 12-48 h at room temperature before released for plasma isolation.

For both cohorts, clinical information was extracted from written/electronic medical records. Only patients who tested positive for SARS-CoV-2 by reverse transcriptase polymerase chain reaction (RT-PCR) on nasopharyngeal/oropharyngeal swabs or sputum were included. Disease severity was scored based on degree of respiratory failure (Mann et al., 2020), as below:

Mild: less than 3 L/min or less than 28% supplemental oxygen and managed in a ward.

Moderate: less than 10 L/min or less than 60% supplemental oxygen, managed in a ward environment; chronic NIV or CPAP (at home) or acute NIV for individuals with COPD.

Severe: any of more than 10 L/min or less than 60% supplemental oxygen, or use of acute NIV, or managed in ICU with invasive ventilation.Where severity of disease changed during admission, the highest disease severity score was selected for classification.

### Methods details

#### Blood plasma isolation

For cohort 1, whole venous blood was collected in tubes containing EDTA (Starstedt) and diluted 1:1 with PBS. Peripheral blood mononuclear cells (PBMCs) were isolated by density gradient centrifugation using Ficoll-Paque Plus (GE Healthcare) and 50 mL SepMate tubes (STEMCELL technologies) according to the manufacturer's protocol. Blood plasma, at the top of the gradient was collected and stored at −80°C until usage. For cohort 2, whole blood was collected in tubes containing EDTA (Starstedt). Blood plasma was collected by centrifugation (1500 g for 10 min at 4°).

#### RNA extraction and miRNA profiling

For Nanostring analyses (Cohort 1), RNA was extracted from 800 μL plasma the Norgen Plasma/Serum RNA Purification Midi kit (Norgen, Cat #56100). A spike-in non-human miRNA (osa-miR-414) was added to each sample (5 μL from a 200 pM per sample) for normalisation. For miRNA profiling we used the Nanostring nCounter™ platform and the human v3 miRNA panel was used containing probes for 827 human miRNAs. Extraction of raw reads and analysis of the individual datasets was carried out using the nSolver™ Analysis Software. For each miRNA the mean plus one standard deviation of negative controls from all counts was subtracted. A minimum value of 1 to all counts. All miRNAs which were expressed above this threshold in fewer than 10% of samples were removed, leaving 280 miRNAs for further analysis. The spike-in count (osa-miR414) was used to normalise miRNA counts. Differential expression analysis on these normalised values was performed by performing Mann Whitney U test, followed by a false discovery rate correction using the Benjamini Hochberg correction to generate adjusted p values.

For cohort 2, RNA was extracted from 200 μL of undiluted plasma using the Norgen Plasma/Serum RNA Purification Mini kit (Norgen, Cat #55000) after addition of the spike RNA at the same concentration as above. Targeted analysis of 9 miRNAs was performed using miRNA Taqman assays (Cat #4427975). Reverse transcription was performed using the Taqman MicroRNA Reverse Transcription Kit (Cat #4366596), by multiplexing primers for all 10 miRNAs (9 miRNAs+spike) after 1 in 6 dilution and using 5 μL RNA as template. Quantitative PCR was performed using a StepOnePlus Real Time PCR System (ThermoFisher) and using Taqman Universal PCR Master Mix (Cat #4305719) and standard cycling conditions as per manufacturer's instructions (Taqman miRNA assays).

#### Cytokine and chemokine profiling

With the exemption of GM-CSF, for cytokine and chemokine profiling bead-based multiplex flow cytometry assays were used. Cytokines and chemokines levels were quantified from patients' plasma fractions using three sets of pre-defined LEGENDplex panels: Human Th-2 cytokine Panel version 2 (IL2, IL4, IL5, IL6, IL9, IL10, IL13, IL17A, IL17F, IL22, IFNγ and TNF), Proinflammatory chemokine Panel 1 (CCL2, CCL3, CCL4, CCL11, CCL17 and CCL20; CXCL1, CXCL5, CXCL8, CXCL9, CXCL10 and CXCL11) plus a separate CCL5 1-plex kit (BioLegend, San Diego, USA) according to manufacturer's instructions. GM-CSF was detected and quantified using a sandwich ABTS ELISA kit (PeproTech EC, Ltd, UK). Statistical significance in differences in cytokine and chemokine expression between groups were assessed using Mann Whitney U test, followed by Benjamini Hochberg correction to generate adjusted p values.

### Quantification and statistical analysis

#### Data integration and statistical methods

Partial least squares regression (PLSR) was performed using the R package ‘pls’ with the response variable encoded as 1.0, 2.0 and 3.0 for mild, moderate and severe observations respectively. Leave-one-patient-out (L-O-P-O) classification was carried out by assigning the class corresponding to the closest integer to the output response. For each patient, a model was built without using the data for any of that patient's samples and the results are given for the prediction of the observations left out for each patient in turn. As results showed mild and moderate patients could not be distinguished, the L-O-P-O classification was repeated using just two classes, with mild and moderate observations combined as one class.

For correspondence analysis (CA), min-max scaling was applied to the data for each chemokine and cytokine so that each variable had a minimum of zero and maximum of 3.0 over all observations. With values less than 1.0 considered as ‘low’, values between 1.0 and 2.0 as ‘medium’ and values >2.0 considered as ‘high’, count data for each severity group was obtained for each variable. To reduce the number of variables, medium counts were excluded from CA, which was performed using the *corresp()* function from the ‘MASS’ package in R. All count data was used in chi-squared tests to assess the significance of any association. Balloon plots were created using only counts for either high values or low values with the function *ballonplot()* from the R package ‘gplot’.

The R package ‘CCA’ was used for canonical correlation analysis (CCA) to find linear combinations of chemokines and cytokines with maximal correlation to a linear combination of the up-regulated miRNAs associated with cell death. To reduce the likelihood of overfitting, we only included cytokines and chemokines with individual correlation to any of these miRNAs >0.4 in absolute value. We developed a model using the 43 samples from 12 patients (chosen randomly from the 17 available observations) with severe disease, using Wilks' Lambda with Rao's F-approximation to assess the significance of correlations. We used the data for the remaining 5 severe patients as an independent test set.

For all correlations between individual variables, Spearman's rank-order correlation was used. Where there were missing values for clinical data, correlations were calculated over all patients for which values were available. Heatmaps showing multiple correlations were created using the package ‘pheatmap’ in R with thresholds for the minimum absolute values shown chosen to limit the size of the heatmaps.

Stepwise logistic regression was also carried out in R using the functions ‘glm’ and ‘step’. Models were compared using the residual deviance (the lack of fit to the perfect model) and Akaike Information Criterion (AIC; a measure of the prediction error). Classification results were obtained by assigning the class with the highest probability.

## Data Availability

• Nanostring miRNA profiling data have been deposited at GEO and are publicly available as of the date of publication. Accession numbers are listed in the key resources table. • This study does not report original code

## Supplemental information

Document S1. Figures S1–S5

Table S1: Cohort 1 characteristics and data: Age, sex, and ethnicity data for the whole cohort and per severity group, related to Figures 1–6

Table S2: Cohort 1 characteristics and data: Clinical measurements for the whole cohort and per severity group, related to Figures 1–6

Table S3: Cohort 1 characteristics and data: Cohort comorbidities and symptoms, related to Figures 1–6

Table S4: Cohort 1 characteristics and data: Sampling information for the whole cohort and per severity group, related to Figures 1–6

Table S5: MiRNA profiling of COVID-19 patients: Severe vs Mild and Moderate, related to Figure 1

Table S6: MiRNA profiling of COVID-19 patients: Mild vs Severe and Moderate, related to Figure 2

Table S7: Cytokine and chemokine profiling of COVID-19 patients. Related to Figure 3

Table S8: Strongest miRNA correlations for severity associated cytokines and chemokines, related to Figure 4

Table S9: Strongest cytokine and chemokine correlations for Cell Death-associated miRNAs, related to Figure 4

Table S10: Cohort 2 (leftover blood sample cohort) characteristics and data, related to Figure 7

Table S11: Forwards stepwise logistic regression miRNA/CC/disease outcome models, related to Figure 7

## References

[bib1] Agarwal V., Bell G.W., Nam J.W., Bartel D.P. (2015). Predicting effective microRNA target sites in mammalian mRNAs. Elife.

[bib2] Ahn S.H., Gu D., Koh Y., Lee H.S., Chi S.W. (2021). AGO CLIP-based imputation of potent siRNA sequences targeting SARS-CoV-2 with antifibrotic miRNA-like activity. Sci. Rep..

[bib3] Aslani M., Mortazavi-Jahromi S.S., Mirshafiey A. (2021). Cytokine storm in the pathophysiology of COVID-19: possible functional disturbances of miRNAs. Int. Immunopharmacol..

[bib4] Bergamaschi L., Mescia F., Turner L., Hanson A.L., Kotagiri P., Dunmore B.J., Ruffieux H., De Sa A., Huhn O., Morgan M.D. (2021). Longitudinal analysis reveals that delayed bystander CD8^+^ T cell activation and early immune pathology distinguish severe COVID-19 from mild disease. Immunity.

[bib5] Bost P., Giladi A., Liu Y., Bendjelal Y., Xu G., David E., Blecher-Gonen R., Cohen M., Medaglia C., Li H. (2020). Host-viral infection maps reveal signatures of severe COVID-19 patients. Cell.

[bib6] Chen G., Wu D., Guo W., Cao Y., Huang D., Wang H., Wang T., Zhang X., Chen H., Yu H. (2020). Clinical and immunological features of severe and moderate coronavirus disease 2019. J. Clin. Invest..

[bib7] Chen N., Zhou M., Dong X., Qu J., Gong F., Han Y., Qiu Y., Wang J., Liu Y., Wei Y. (2020). Epidemiological and clinical characteristics of 99 cases of 2019 novel coronavirus pneumonia in Wuhan, China: a descriptive study. Lancet.

[bib8] Chi Y., Ge Y., Wu B., Zhang W., Wu T., Wen T., Liu J., Guo X., Huang C., Jiao Y. (2020). Serum cytokine and chemokine profile in relation to the severity of coronavirus disease 2019 in China. J. Infect. Dis..

[bib9] Chua R.L., Lukassen S., Trump S., Hennig B.P., Wendisch D., Pott F., Debnath O., Thurmann L., Kurth F., Volker M.T. (2020). COVID-19 severity correlates with airway epithelium-immune cell interactions identified by single-cell analysis. Nat. Biotechnol..

[bib10] COvid-19 Multi-omics Blood ATlas (COMBAT) Consortium (2021). A blood atlas of COVID-19 defines hallmarks of disease severity and specificity. MedRxiv.

[bib11] Coperchini F., Chiovato L., Rotondi M. (2021). Interleukin-6, CXCL10 and infiltrating macrophages in COVID-19-related cytokine storm: not one for all but all for one!. Front. Immunol..

[bib12] Correia C.N., Nalpas N.C., McLoughlin K.E., Browne J.A., Gordon S.V., MacHugh D.E., Shaughnessy R.G. (2017). Circulating microRNAs as potential biomarkers of infectious disease. Front. Immunol..

[bib13] Czajka P., Fitas A., Jakubik D., Eyileten C., Gasecka A., Wicik Z., Siller-Matula J.M., Filipiak K.J., Postula M. (2021). MicroRNA as potential biomarkers of platelet function on antiplatelet therapy: a review. Front. Physiol..

[bib14] Dash S., Dash C., Pandhare J. (2021). Therapeutic significance of microRNA-mediated regulation of PARP-1 in SARS-CoV-2 infection. Noncoding RNA.

[bib15] de Gonzalo-Calvo D., Benitez I.D., Pinilla L., Carratala A., Moncusi-Moix A., Gort-Paniello C., Molinero M., Gonzalez J., Torres G., Bernal M. (2021). Circulating microRNA profiles predict the severity of COVID-19 in hospitalized patients. Transl. Res..

[bib16] Del Valle D.M., Kim-Schulze S., Huang H.H., Beckmann N.D., Nirenberg S., Wang B., Lavin Y., Swartz T.H., Madduri D., Stock A. (2020). An inflammatory cytokine signature predicts COVID-19 severity and survival. Nat. Med..

[bib17] Diao B., Wang C., Tan Y., Chen X., Liu Y., Ning L., Chen L., Li M., Liu Y., Wang G. (2020). Reduction and functional exhaustion of T cells in patients with coronavirus disease 2019 (COVID-19). Front. Immunol..

[bib18] Duecker R.P., Adam E.H., Wirtz S., Gronau L., Khodamoradi Y., Eberhardt F.J., Donath H., Gutmann D., Vehreschild M., Zacharowski K. (2021). The MiR-320 family is strongly downregulated in patients with COVID-19 induced severe respiratory failure. Int. J. Mol. Sci..

[bib19] Esai Selvan M. (2020). Risk factors for death from COVID-19. Nat. Rev. Immunol..

[bib20] Farr R.J., Rootes C.L., Rowntree L.C., Nguyen T.H.O., Hensen L., Kedzierski L., Cheng A.C., Kedzierska K., Au G.G., Marsh G.A. (2021). Altered microRNA expression in COVID-19 patients enables identification of SARS-CoV-2 infection. PLoS Pathog..

[bib21] Feketea G.M., Vlacha V. (2020). The diagnostic significance of usual biochemical parameters in coronavirus disease 19 (COVID-19): albumin to globulin ratio and CRP to albumin ratio. Front. Med. (Lausanne).

[bib22] Galvan-Roman J.M., Rodriguez-Garcia S.C., Roy-Vallejo E., Marcos-Jimenez A., Sanchez-Alonso S., Fernandez-Diaz C., Alcaraz-Serna A., Mateu-Albero T., Rodriguez-Cortes P., Sanchez-Cerrillo I. (2021). IL-6 serum levels predict severity and response to tocilizumab in COVID-19: an observational study. J. Allergy Clin. Immunol..

[bib23] Gambardella J., Coppola A., Izzo R., Fiorentino G., Trimarco B., Santulli G. (2021). Role of endothelial miR-24 in COVID-19 cerebrovascular events. Crit. Care.

[bib24] Gambardella J., Sardu C., Morelli M.B., Messina V., Castellanos V., Marfella R., Maggi P., Paolisso G., Wang X., Santulli G. (2021). Exosomal microRNAs Drive Thrombosis in COVID-19. MedRxiv.

[bib25] Gasparello J., d'Aversa E., Breveglieri G., Borgatti M., Finotti A., Gambari R. (2021). In vitro induction of interleukin-8 by SARS-CoV-2 Spike protein is inhibited in bronchial epithelial IB3-1 cells by a miR-93-5p agomiR. Int. Immunopharmacol..

[bib26] Henry B., Cheruiyot I., Vikse J., Mutua V., Kipkorir V., Benoit J., Plebani M., Bragazzi N., Lippi G. (2020). Lymphopenia and neutrophilia at admission predicts severity and mortality in patients with COVID-19: a meta-analysis. Acta Biomed..

[bib27] Heuberger J., Trimpert J., Vladimirova D., Goosmann C., Lin M., Schmuck R., Mollenkopf H.J., Brinkmann V., Tacke F., Osterrieder N., Sigal M. (2021). Epithelial response to IFN-gamma promotes SARS-CoV-2 infection. EMBO Mol. Med..

[bib28] Houshmandfar S., Saeedi-Boroujeni A., Rashno M., Khodadadi A., Mahmoudian-Sani M.R. (2021). miRNA-223 as a regulator of inflammation and NLRP3 inflammasome, the main fragments in the puzzle of immunopathogenesis of different inflammatory diseases and COVID-19. Naunyn Schmiedebergs Arch. Pharmacol..

[bib29] Hue S., Beldi-Ferchiou A., Bendib I., Surenaud M., Fourati S., Frapard T., Rivoal S., Razazi K., Carteaux G., Delfau-Larue M.H. (2020). Uncontrolled innate and impaired adaptive immune responses in patients with COVID-19 acute respiratory distress syndrome. Am. J. Respir. Crit. Care Med..

[bib30] Hum C., Loiselle J., Ahmed N., Shaw T.A., Toudic C., Pezacki J.P. (2021). MicroRNA mimics or inhibitors as antiviral therapeutic approaches against COVID-19. Drugs.

[bib31] Ito T., Carson W.F., Cavassani K.A., Connett J.M., Kunkel S.L. (2011). CCR6 as a mediator of immunity in the lung and gut. Exp. Cell Res..

[bib32] Karki R., Sharma B.R., Tuladhar S., Williams E.P., Zalduondo L., Samir P., Zheng M., Sundaram B., Banoth B., Malireddi R.K.S. (2021). Synergism of TNF-alpha and IFN-gamma triggers inflammatory cell death, tissue damage, and mortality in SARS-CoV-2 infection and cytokine shock syndromes. Cell.

[bib33] Khokhar M., Tomo S., Purohit P. (2022). MicroRNAs based regulation of cytokine regulating immune expressed genes and their transcription factors in COVID-19. Meta Gene.

[bib34] Laing A.G., Lorenc A., Del Molino Del Barrio I., Das A., Fish M., Monin L., Munoz-Ruiz M., McKenzie D.R., Hayday T.S., Francos-Quijorna I. (2020). A dynamic COVID-19 immune signature includes associations with poor prognosis. Nat. Med..

[bib35] Li J., Han X., Wan Y., Zhang S., Zhao Y., Fan R., Cui Q., Zhou Y. (2018). TAM 2.0: tool for MicroRNA set analysis. Nucleic Acids Res..

[bib36] Li J., Rong L., Cui R., Feng J., Jin Y., Chen X., Xu R. (2021). Dynamic changes in serum IL-6, IL-8, and IL-10 predict the outcome of ICU patients with severe COVID-19. Ann. Palliat. Med..

[bib37] Lippi G., Plebani M., Henry B.M. (2020). Thrombocytopenia is associated with severe coronavirus disease 2019 (COVID-19) infections: a meta-analysis. Clin. Chim. Acta.

[bib38] Liu C., Martins A.J., Lau W.W., Rachmaninoff N., Chen J., Imberti L., Mostaghimi D., Fink D.L., Burbelo P.D., Dobbs K. (2021). Time-resolved systems immunology reveals a late juncture linked to fatal COVID-19. Cell.

[bib39] Liu X., Wang H., Shi S., Xiao J. (2021). Association between IL-6 and severe disease and mortality in COVID-19 disease: a systematic review and meta-analysis. Postgrad. Med. J..

[bib40] Lucas C., Wong P., Klein J., Castro T.B.R., Silva J., Sundaram M., Ellingson M.K., Mao T., Oh J.E., Israelow B. (2020). Longitudinal analyses reveal immunological misfiring in severe COVID-19. Nature.

[bib41] Mann E.R., Menon M., Knight S.B., Konkel J.E., Jagger C., Shaw T.N., Krishnan S., Rattray M., Ustianowski A., Bakerly N.D. (2020). Longitudinal immune profiling reveals key myeloid signatures associated with COVID-19. Sci. Immunol..

[bib42] Matarese A., Gambardella J., Sardu C., Santulli G. (2020). miR-98 regulates TMPRSS2 expression in human endothelial cells: key implications for COVID-19. Biomedicines.

[bib43] McDonald J.T., Enguita F.J., Taylor D., Griffin R.J., Priebe W., Emmett M.R., Sajadi M.M., Harris A.D., Clement J., Dybas J.M. (2021). Role of miR-2392 in driving SARS-CoV-2 infection. Cell Rep..

[bib44] Mitchell A.J., Gray W.D., Hayek S.S., Ko Y.A., Thomas S., Rooney K., Awad M., Roback J.D., Quyyumi A., Searles C.D. (2016). Platelets confound the measurement of extracellular miRNA in archived plasma. Sci. Rep..

[bib45] Mone P., Gambardella J., Wang X., Jankauskas S.S., Matarese A., Santulli G. (2021). miR-24 targets the transmembrane glycoprotein neuropilin-1 in human brain microvascular endothelial cells. Noncoding RNA.

[bib46] Montalvo Villalba M.C., Valdes Ramirez O., Mune Jimenez M., Arencibia Garcia A., Martinez Alfonso J., Gonzalez Baez G., Roque Arrieta R., Rosell Simon D., Alvarez Gainza D., Sierra Vazquez B. (2020). Interferon gamma, TGF-beta1 and RANTES expression in upper airway samples from SARS-CoV-2 infected patients. Clin. Immunol..

[bib47] Park J.H., Choi Y., Lim C.W., Park J.M., Yu S.H., Kim Y., Han H.J., Kim C.H., Song Y.S., Kim C. (2021). Potential therapeutic effect of micrornas in extracellular vesicles from mesenchymal stem cells against SARS-CoV-2. Cells.

[bib48] Plowman T., Lagos D. (2021). Non-coding RNAs in COVID-19: emerging insights and current questions. Noncoding RNA.

[bib49] Rampotas A., Pavord S. (2020). Platelet aggregates, a marker of severe COVID-19 disease. J. Clin. Pathol..

[bib50] Sabbatinelli J., Giuliani A., Matacchione G., Latini S., Laprovitera N., Pomponio G., Ferrarini A., Svegliati Baroni S., Pavani M., Moretti M. (2021). Decreased serum levels of the inflammaging marker miR-146a are associated with clinical non-response to tocilizumab in COVID-19 patients. Mech. Ageing Dev..

[bib51] Saris A., Reijnders T.D.Y., Reijm M., Hollander J.C., de Buck K., Schuurman A.R., Duitman J., Heunks L., Aman J., Bogaard H.J. (2021). Enrichment of CCR6(+) CD8(+) T cells and CCL20 in the lungs of mechanically ventilated patients with COVID-19. Eur. J. Immunol..

[bib52] Schulte-Schrepping J., Reusch N., Paclik D., Bassler K., Schlickeiser S., Zhang B., Kramer B., Krammer T., Brumhard S., Bonaguro L. (2020). Severe COVID-19 is marked by a dysregulated myeloid cell compartment. Cell.

[bib53] Sedgwick P. (2014). Bias in observational study designs: prospective cohort studies. BMJ.

[bib54] Sohel M.M.H. (2020). Circulating microRNAs as biomarkers in cancer diagnosis. Life Sci..

[bib55] Stephenson E., Reynolds G., Botting R.A., Calero-Nieto F.J., Morgan M.D., Tuong Z.K., Bach K., Sungnak W., Worlock K.B., Yoshida M. (2021). Single-cell multi-omics analysis of the immune response in COVID-19. Nat. Med..

[bib56] Terpos E., Ntanasis-Stathopoulos I., Elalamy I., Kastritis E., Sergentanis T.N., Politou M., Psaltopoulou T., Gerotziafas G., Dimopoulos M.A. (2020). Hematological findings and complications of COVID-19. Am. J. Hematol..

[bib57] Thwaites R.S., Sanchez Sevilla Uruchurtu A., Siggins M.K., Liew F., Russell C.D., Moore S.C., Fairfield C., Carter E., Abrams S., Short C.E. (2021). Inflammatory profiles across the spectrum of disease reveal a distinct role for GM-CSF in severe COVID-19. Sci. Immunol..

[bib58] Vanderbeke L., Van Mol P., Van Herck Y., De Smet F., Humblet-Baron S., Martinod K., Antoranz A., Arijs I., Boeckx B., Bosisio F.M. (2021). Monocyte-driven atypical cytokine storm and aberrant neutrophil activation as key mediators of COVID-19 disease severity. Nat. Commun..

[bib59] Wang E.Y., Mao T., Klein J., Dai Y., Huck J.D., Jaycox J.R., Liu F., Zhou T., Israelow B., Wong P. (2021). Diverse functional autoantibodies in patients with COVID-19. Nature.

[bib60] Wittmann J., Jack H.M. (2010). Serum microRNAs as powerful cancer biomarkers. Biochim. Biophys. Acta.

[bib61] Woodruff M.C., Ramonell R.P., Nguyen D.C., Cashman K.S., Saini A.S., Haddad N.S., Ley A.M., Kyu S., Howell J.C., Ozturk T. (2020). Extrafollicular B cell responses correlate with neutralizing antibodies and morbidity in COVID-19. Nat. Immunol..

[bib62] Wu F., Zhao S., Yu B., Chen Y.M., Wang W., Song Z.G., Hu Y., Tao Z.W., Tian J.H., Pei Y.Y. (2020). A new coronavirus associated with human respiratory disease in China. Nature.

[bib63] Zeng L., Cui J., Wu H., Lu Q. (2014). The emerging role of circulating microRNAs as biomarkers in autoimmune diseases. Autoimmunity.

[bib64] Zhao Y., Qin L., Zhang P., Li K., Liang L., Sun J., Xu B., Dai Y., Li X., Zhang C. (2020). Longitudinal COVID-19 profiling associates IL-1RA and IL-10 with disease severity and RANTES with mild disease. JCI Insight.

