# Supplementary material for: Integrated miRNA/cytokine/chemokine profiling reveals severity-associated step changes and principal correlates of fatality in COVID-19
Source: iScience. 2021 Dec 20;25(1):103672. doi: 10.1016/j.isci.2021.103672 (PMC8686203; doi:10.1016/j.isci.2021.103672)
Supplement: Document S1. Figures S1–S5 [file mmc1.pdf]

iScience, Volume *25*

Supplemental information

Integrated miRNA/cytokine/chemokine profiling reveals severity-associated step changes and principal correlates of fatality in COVID-19

Julie C. Wilson, David Kealy, Sally R. James, Tobias Plowman, Katherine Newling, Christopher Jagger, Kara Filbey, Elizabeth R. Mann, Joanne E. Konkel, Madhvi Menon, Sean B. Knight, Angela Simpson, CIRCO Collaborative Group, Aliya Prihartadi, Greg Forshaw, Neil Todd, David R.A. Yates, John R. Grainger, Tracy Hussell, Paul M. Kaye, Nathalie Signoret, and Dimitris Lagos

Supplemental Figure S1 (related to Figures 1-6): Study design and miRNA profiling of hospitalised patients with COVID-19

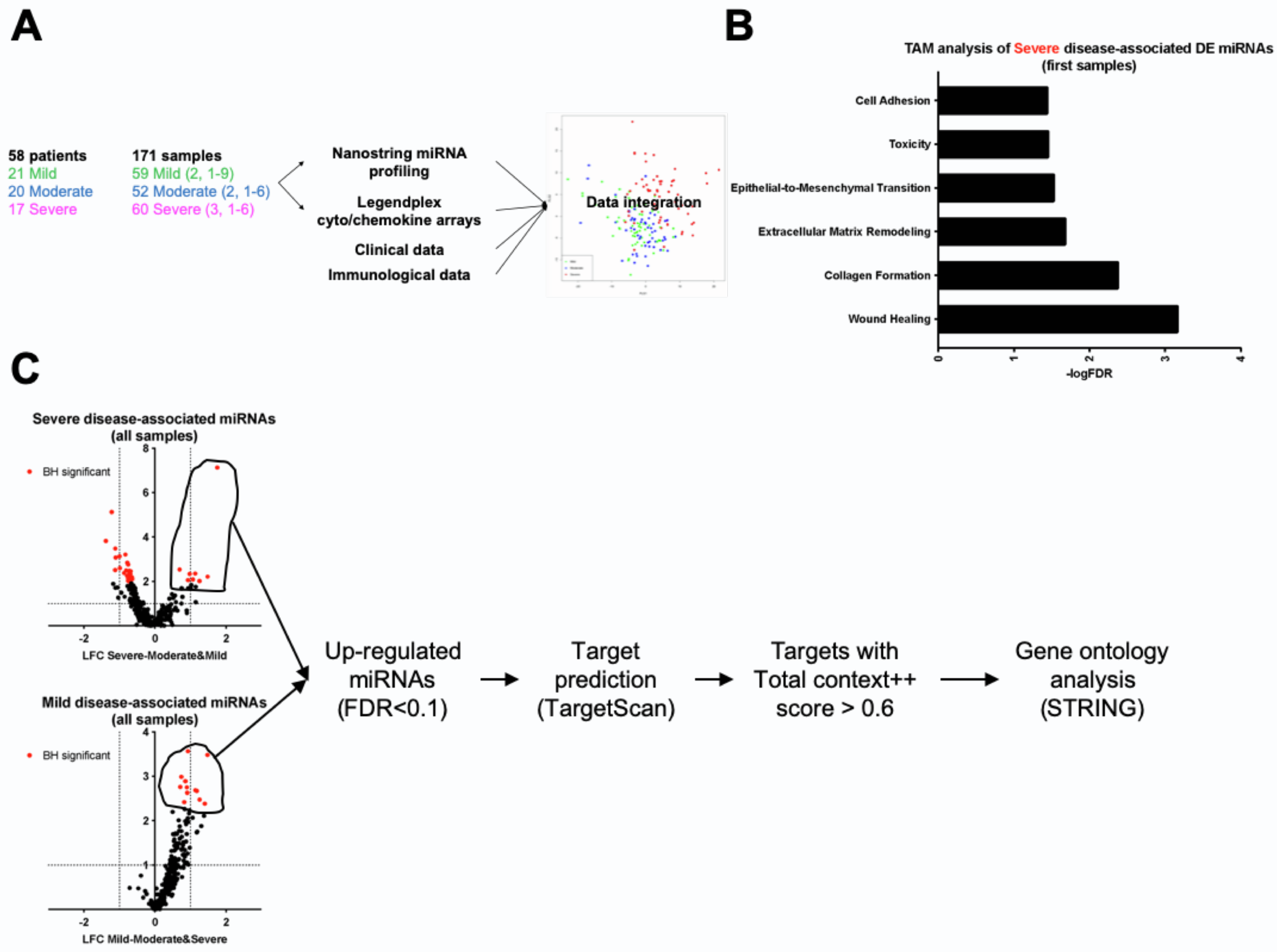

Figure S1 (related to Figures 1-6): Study design and miRNA profiling of hospitalised patients with COVID-19

A. Study schematic overview.

B. Significantly over-represented functional terms within DE miRNAs in severe cases, including only first samples (corresponding to Figure 1C).

C. Schematic outlining selection of top predicted targets of miRNAs up-regulated in severe or mild COVID-19 samples. TargetScan was used to identify all targets for all selected miRNAs (circled in the volcano plots) and high confidence targets (Total context++ score > 0.6) were selected for gene ontology analysis.

Supplemental Figure S2 (related to Figure 3): Cytokine and chemokine signatures of COVID-19

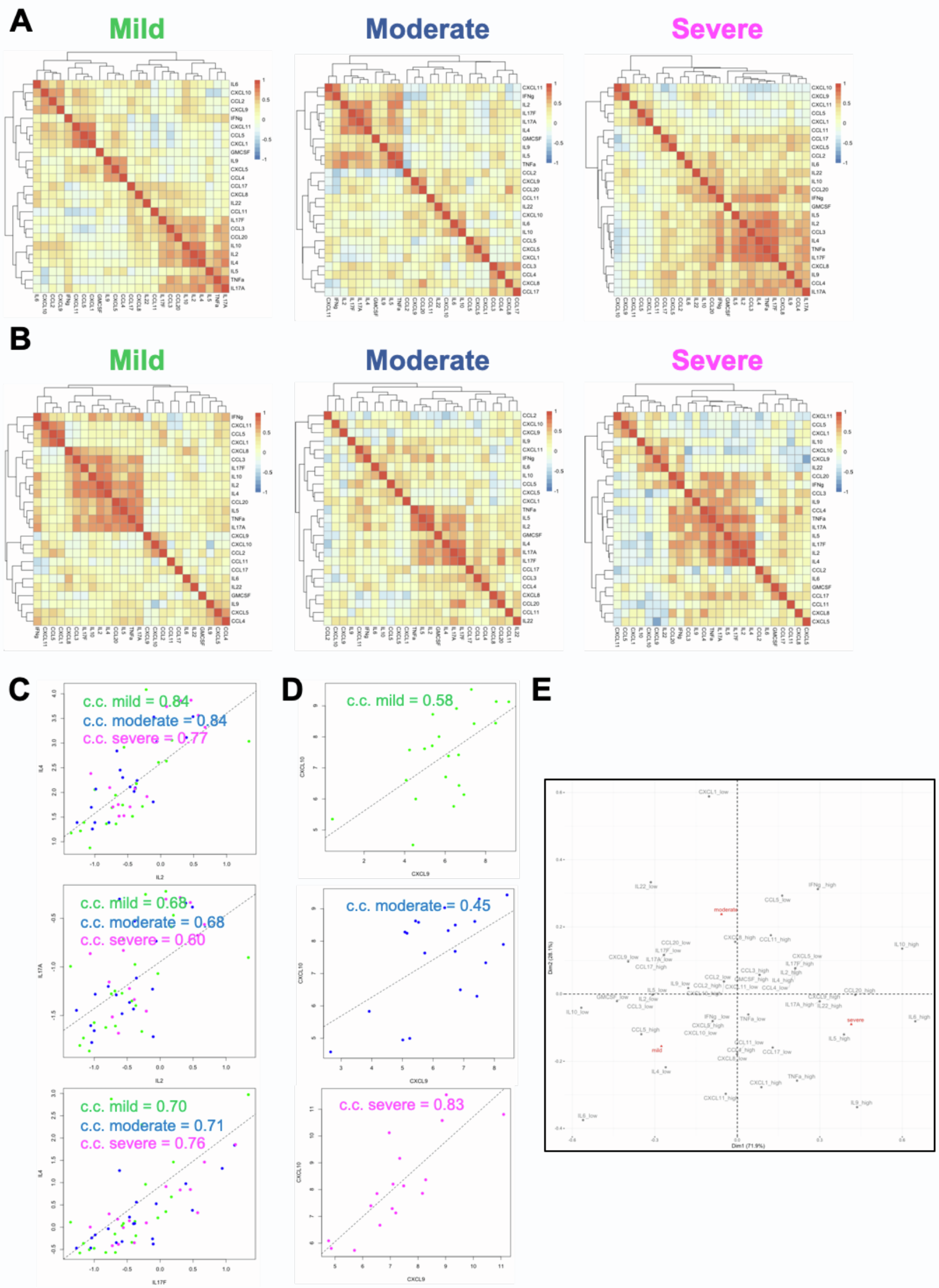

Figure S2 (related to Figure 3): Cytokine and chemokine signatures of COVID-19

A. Heatmaps showing Spearman correlation coefficients (c.c.) between cytokines and chemokines for mild, moderate, and severe groups, using all samples individually.

B. As in A, but only using measurements from first available samples for each patient.

C.Plots of IL2 against IL4, IL17A, and IL17F levels in mild (green), moderate (blue), and severe (magenta) patients. Correlation coefficients shown at the top of each dot plot.

D. Plots of CXCL9 against CXCL10 levels in mild (green), moderate (blue), and severe (magenta) patients. Correlation coefficients (c.c.) also shown.

E. Correspondence analysis for cytokines and chemokines showing the association between severity groups and high and low values of cytokines and chemokines.

Supplemental Figure S3 (related to Figure 4): Integration of miRNA, cytokine, and chemokine signatures

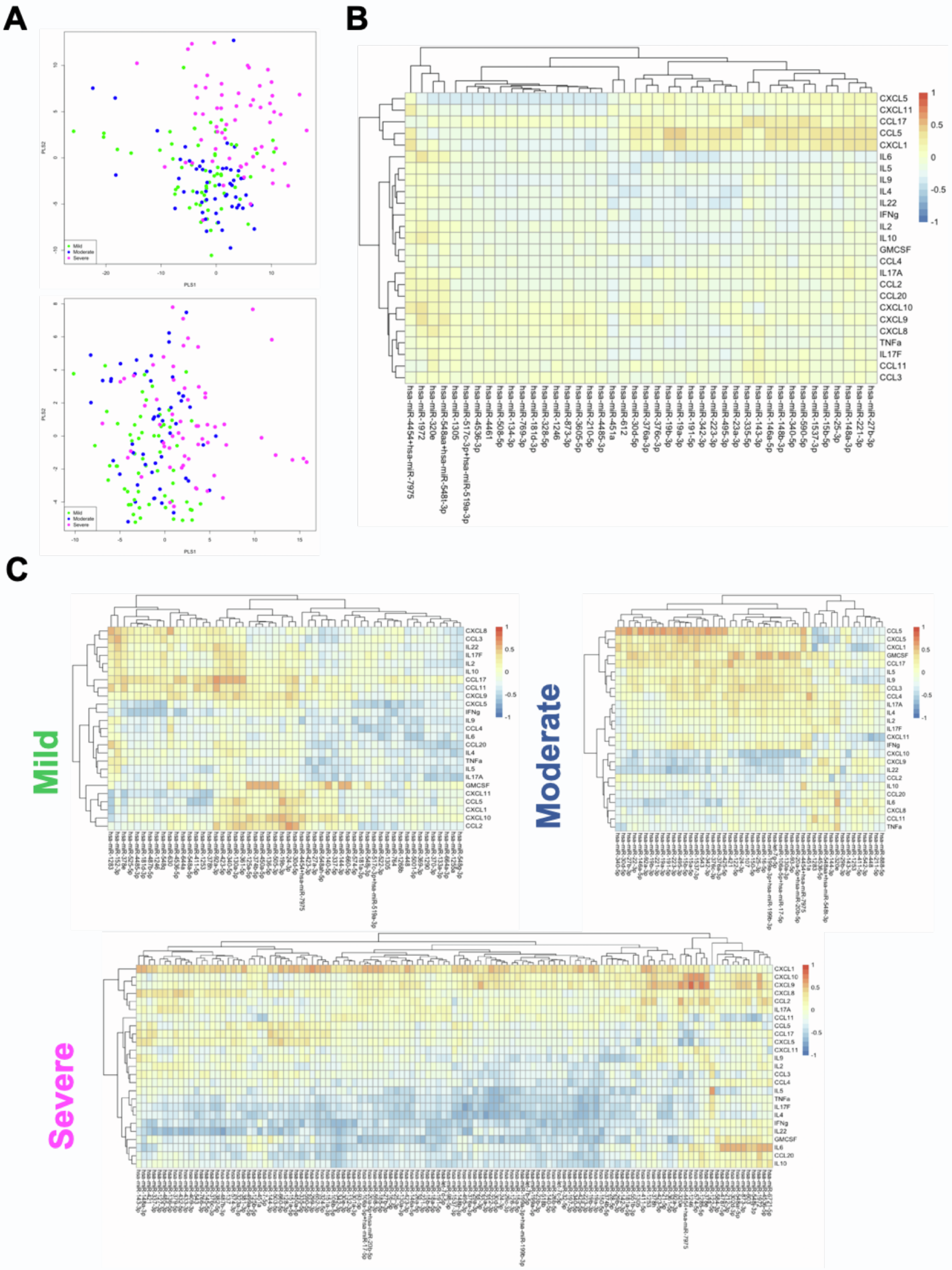

Figure S3 (related to Figure 4): Integration of miRNA, cytokine, and chemokine signatures

A. Scores plots for the first two latent variables from partial least squares regression (PLSR) obtained using DE miRNA measurements (left) or cytokine and chemokine values (right), coloured by severity group (mild, moderate and severe).

B. Heatmaps showing Spearman correlation coefficients (c.c.) between miRNAs and cytokines and chemokines. The miRNAs shown have a correlation greater than 0.3 in absolute value with at least one cytokine or chemokine. No values reach absolute c.c. of 0.4. Correlations are calculated over all severity groups after averaging values for any timepoints available for each patient.

C. Heatmaps showing Spearman correlation coefficients (c.c.) between miRNAs and cytokines and chemokines for mild, moderate, and severe groups. For each patient, data for all available timepoints are averaged. All miRNAs with c.c. > 0.5 in absolute value with any cytokine or chemokine are included.

Supplemental Figure S4 (related to Figure 6): Top correlated miRNAs with clinical parameters

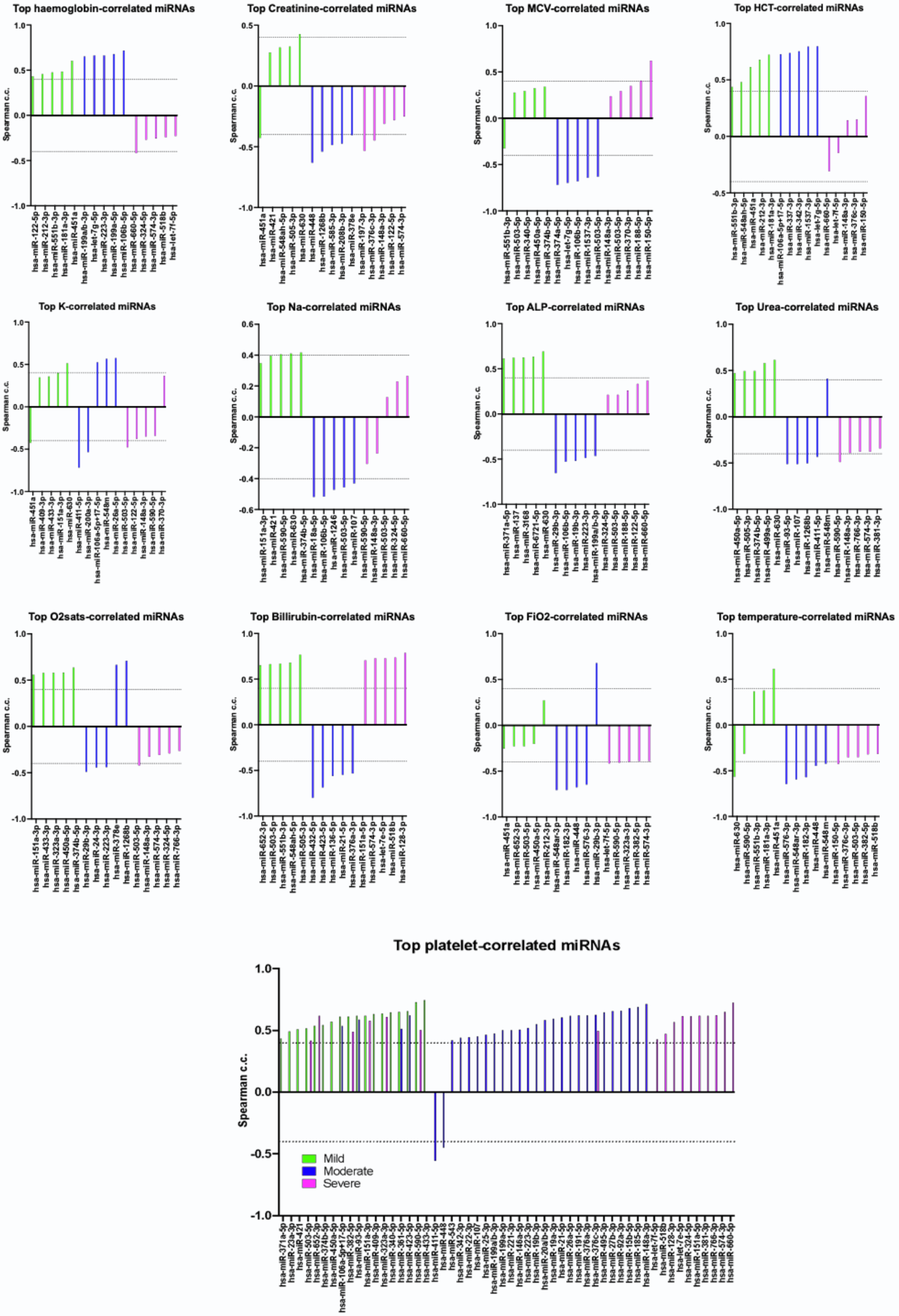

Figure S4 (related to Figure 6): Top correlated miRNAs with clinical parameters
Spearman correlation coefficients for top 5 correlated miRNAs with indicated clinical parameters in mild (green), moderate (blue), and severe (magenta) groups.

Supplemental Figure S5 (related to Figure 7): Correlations between cytokines and chemokines in the leftover blood sample cohort

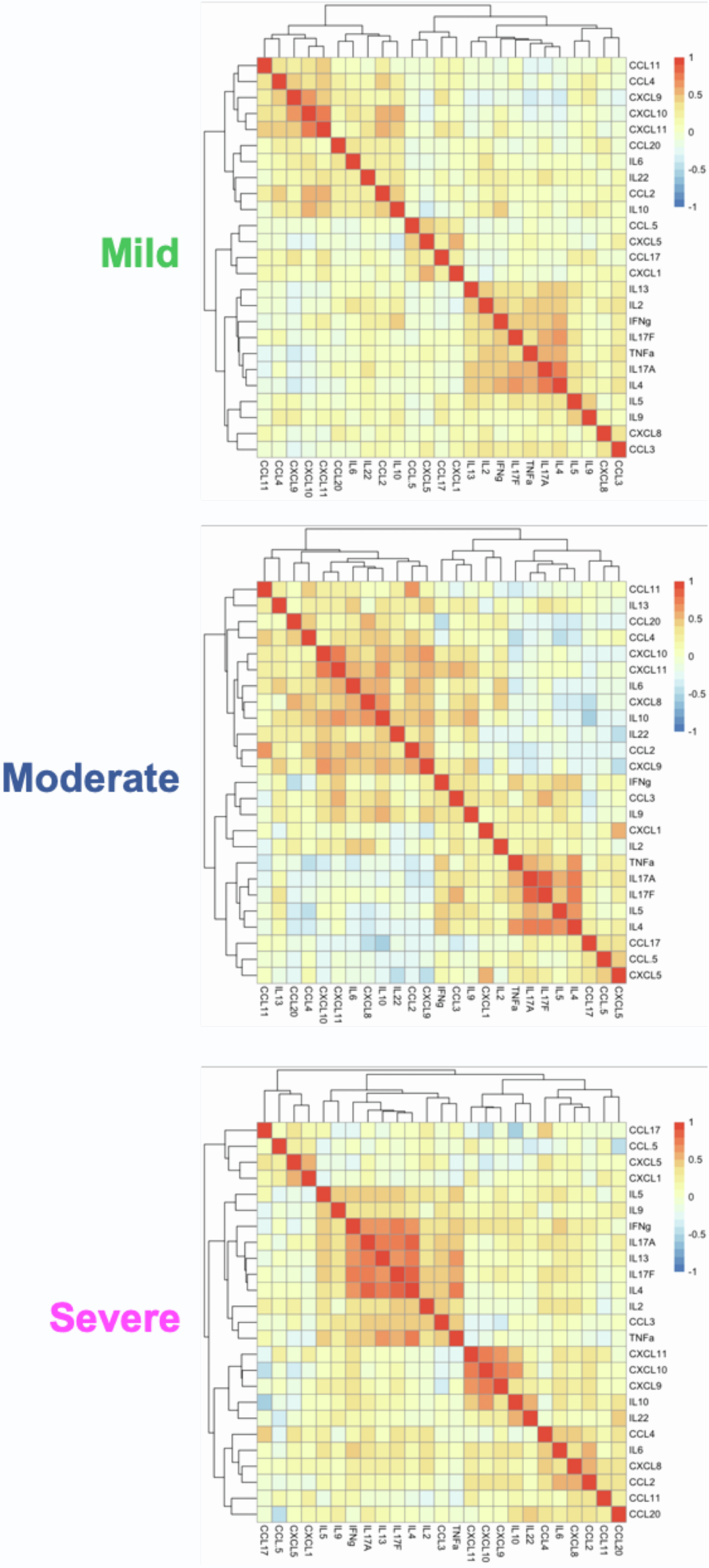

Figure S5 (related to Figure 7): Correlations between cytokines and chemokines in the leftover blood sample cohort
Heatmaps showing Spearman correlation coefficients (c.c.) between cytokines and chemokines for mild, moderate, and severe groups, in the leftover blood sample cohort.
